# Identification of Sclareol As a Natural Neuroprotective Ca_v_1.3‐Antagonist Using Synthetic Parkinson‐Mimetic Gene Circuits and Computer‐Aided Drug Discovery

**DOI:** 10.1002/advs.202102855

**Published:** 2022-01-18

**Authors:** Hui Wang, Mingqi Xie, Giorgio Rizzi, Xin Li, Kelly Tan, Martin Fussenegger

**Affiliations:** ^1^ Department of Biosystems Science and Engineering ETH Zurich Mattenstrasse 26 Basel 4058 Switzerland; ^2^ Biozentrum University of Basel Klingelbergstrasse 50/70 Basel 4056 Switzerland; ^3^ University of Basel Faculty of Science Mattenstrasse 26 Basel CH‐4058 Switzerland; ^4^ Present address: Lonza AG Lonzastrasse Visp 3930 Switzerland; ^5^ Present address: Key Laboratory of Growth Regulation and Translational Research of Zhejiang Province School of Life Sciences Westlake University Shilongshan Road 18 Hangzhou P. R. China; ^6^ Present address: Inscopix Inc Embarcadero Way Palo Alto CA 94303 USA

**Keywords:** drug discovery, neuroprotection, Parkinson's disease, sclareol, synthetic biology, voltage‐gated calcium channels

## Abstract

Parkinson's disease (PD) results from selective loss of substantia nigra dopaminergic (SNc DA) neurons, and is primarily caused by excessive activity‐related Ca^2+^ oscillations. Although L‐type voltage‐gated calcium channel blockers (CCBs) selectively inhibiting Ca_v_1.3 are considered promising candidates for PD treatment, drug discovery is hampered by the lack of high‐throughput screening technologies permitting isoform‐specific assessment of Cav‐antagonistic activities. Here, a synthetic‐biology‐inspired drug‐discovery platform enables identification of PD‐relevant drug candidates. By deflecting Cav‐dependent activation of nuclear factor of activated T‐cells (NFAT)‐signaling to repression of reporter gene translation, they engineered a cell‐based assay where reporter gene expression is activated by putative CCBs. By using this platform in combination with in silico virtual screening and a trained deep‐learning neural network, sclareol is identified from a essential oils library as a structurally distinctive compound that can be used for PD pharmacotherapy. In vitro studies, biochemical assays and whole‐cell patch‐clamp recordings confirmed that sclareol inhibits Ca_v_1.3 more strongly than Ca_v_1.2 and decreases firing responses of SNc DA neurons. In a mouse model of PD, sclareol treatment reduced DA neuronal loss and protected striatal network dynamics as well as motor performance. Thus, sclareol appears to be a promising drug candidate for neuroprotection in PD patients.

## Graphical abstract

High‐throughput, multiplexed quantification of Ca_V_‐mediated cell responses using synthetic gene circuits combined with in silico virtual screening and deep‐learning‐based prediction identified sclareol as a novel Ca_v_1.3‐antagonist that could evolve as neuroprotective pharmacotherapy of Parkinson's disease in the future.

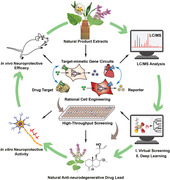

## 1 Introduction

Parkinson's disease (PD) is an age‐related neurodegenerative disorder characterized by progressive motor impairments such as tremors, rigidity, and bradykinesia.^[^ ^1^, ^2^, ^3^ ^]^ These symptoms are primarily driven by the selective loss of mesencephalic dopamine‐producing neurons in the pars compacta of the substantia nigra (SNc) of the midbrain.^[^ ^1^, ^2^, ^3^, ^4^, ^5^ ^]^ Defective dopaminergic (DA) neurons are characterized by the formation of Lewy bodies consisting of ubiquitin and *α*‐synuclein aggregates, are highly sensitive to stress, and show markedly impaired mitochondrial functions, which lead to reduced ATP production and poor calcium homeostasis.^[^ ^3^, ^6^ ^]^ Excessive‐activity‐related Ca^2+^ oscillations contribute to the generation of reactive oxygen species (ROS), resulting in excitotoxicity and apoptosis.^[^ ^5^, ^7^ ^]^ Although a plethora of putative therapeutics have been proposed to relieve PD symptoms, including DA receptor agonists, anti‐inflammatory drugs, inhibitors of *α*‐synuclein aggregates, neurotrophic growth factors, and calpain inhibitors, none of them affords fully effective neuroprotection—a term that, strictly defined, refers to any treatment strategy that protects the integrity of DA neurons.^[^ ^3^ ^]^

In recent years, strategies based on inhibition of L‐type voltage‐gated calcium channels (LTCC) with dihydropyridine (DHP) blockers have gained increased attention for neuroprotective therapy of PD.^[^ ^8^, ^9^ ^]^ Specifically, Ca_V_1.3 is an LTCC isoform primarily expressed in neurons and pancreatic endocrine cells, and opens at subthreshold membrane potentials due to its negative activation voltage range.^[^ ^10^, ^11^ ^]^ DA neurons exhibit Ca_V_1.3‐dependent pace‐making activity, which is a major contributor to excitotoxicity during PD progression.^[^ ^5^, ^9^ ^]^ However, DHP channel blockers are generally non‐selective, blocking both Ca_V_1.2 and Ca_V_1.3 channel isoforms in most cases.^[^ ^12^ ^]^ Because Ca_V_1.2 channels are expressed at very high levels in cardiac tissues, cross‐antagonism to these channels severely limits the dose of DHPs that can be used for neuroprotective purposes.^[^ ^7^, ^12^ ^]^ Therefore, Ca_V_1.3‐selective blockers without Ca_V_1.2‐mediated cardiovascular side effects are currently considered elusive candidates for PD drug discovery.^[^ ^9^ ^]^

Here, by capitalizing on synthetic biology‐inspired gene circuits that can flexibly program cells to perform application‐specific biological tasks with high robustness and precision,^[^ ^13^ ^]^ we have custom‐designed a mammalian cell‐based drug discovery platform for high‐throughput screening (HTS) of isoform‐specific calcium channel blockers (CCBs). Specifically, deflection of Ca_V_‐dependent NFAT‐activation to the repression of reporter protein translation allowed for the engineering of an antagonist‐inducible reporter system (CaB‐A assay) that effectively reduced the susceptibility to false‐negatives associated with cytotoxicity‐mediated signal decrease. After validating this technology with a selection of clinically approved CCB drugs, we identified five plant‐derived essential oils that could effectively block Ca_v_1.2 and Ca_v_1.3. Further integration of in silico virtual screening and deep‐learning technology eventually enabled the identification of sclareol—an essential constituent of the long‐established Mediterranean medicinal herb *Salvia sclarea*—as a most relevant bioactive compound that inhibits Ca_v_1.3 more strongly than Ca_v_1.2. Finally, we demonstrated neuroprotective activities of sclareol both in vitro and in vivo. Using whole cell patch‐clamp recordings, we provide evidence that sclareol decreases excessive neuronal activity of substantia nigra dopaminergic (SNc DA) cells. Furthermore, we show that sclareol reduces SNc DA neuronal degeneration in a mouse model of PD and protects striatal cellular network dynamics and motor performance, as compared to vehicle‐treated mice. Thus, sclareol appears to be a promising lead compound/candidate drug for neuroprotection in PD patients. We believe the combined application of synthetic‐biology‐inspired technology, advanced computational methods, and molecular medicine, as exemplified here, represents an efficient platform that could help to set the stage for next‐generation drug discovery in a variety of contexts.

## 2 Results

### 2.1 Engineering of a Cell‐Based Drug Screening Platform for Multiplexed and Use‐Dependent Analysis of Ca_V_1 Channel Blockers

**Figure 1:**
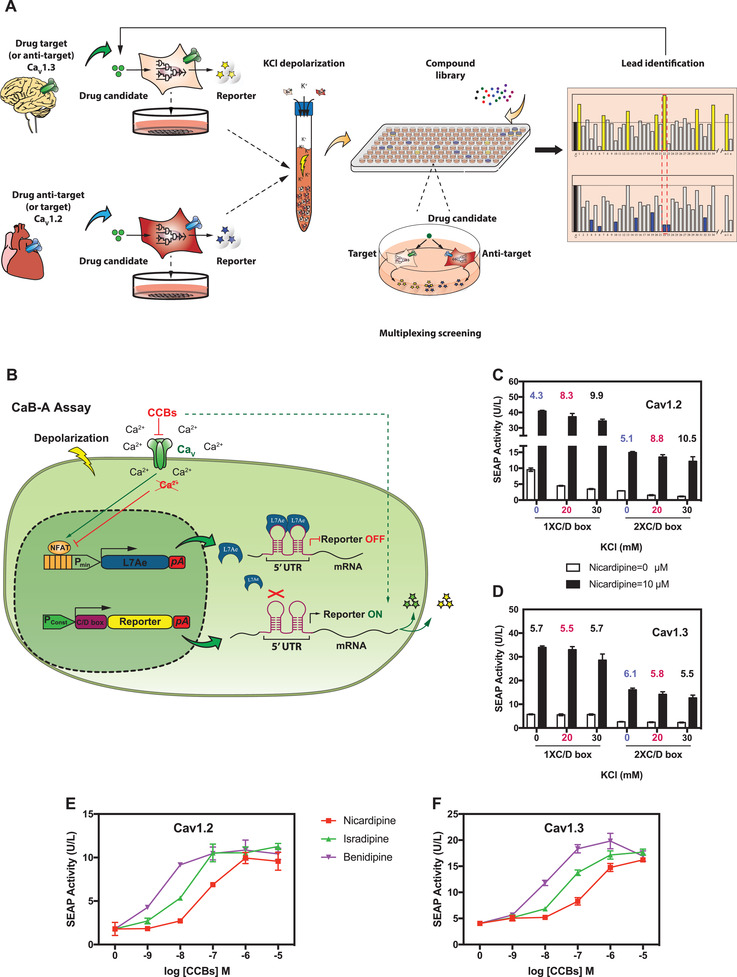
Development of a cell‐based assay for multiplexed drug screening. A) Experimental setup. For multiplexed screening of anti‐Parkinson drugs, Ca_V_1.3 (target)‐specific and Ca_V_1.2 (anti‐target)‐specific cell populations—each controlling the expression of a flexibly chosen reporter protein—are placed in the same reaction well to enable simultaneous assessment of inhibitory potency and specificity in potassium chloride (KCl)‐mediated cell depolarization. B) Design of a CCB‐activated (CaB‐A) reporter assay. Synthetic NFAT‐specific promoters control the production of L7Ae, which inhibits the translation of reporter mRNA by binding to specific C/D‐box aptamers in the 5’‐UTR. CCBs activate reporter protein expression by inhibiting depolarization‐dependent L7Ae expression. C,D) Optimization of CaB‐A for use‐dependent CCB analysis. (C) Ca_V_1.2 (pCa_V_1.2/pKK56)‐ or (D) Ca_V_1.3 (pCa_V_1.3/pKK56)‐transgenic HEK‐293 cells were co‐transfected with a NFAT‐controlled L7Ae expression vector (pMX125; P_NFAT4_‐L7Ae‐pA) and different reporter vectors containing one (pMX195; P_SV40_‐(C/D‐box)_1_‐SEAP‐pA) or two tandem C/D‐box aptamer repeats (pMX199; P_SV40_‐(C/D‐box)_2_‐SEAP‐pA). The cells were depolarized with different levels of KCl (0, 20, and 30 mm) and cultivated for 48 h in the absence or presence (10 µm) of nicardipine. SEAP levels in culture supernatants were scored. Data points are presented as mean ± SD (n = 3 independent experiments). Numerical values displayed on top of each column‐group represent induction‐folds, calculated as the SEAP values resulting from 10 µm nicardipine divided by SEAP values resulting from 0 µm nicardipine. (E, F) Validation of CaB‐A with clinically approved CCBs. E) HEK‐293 cells transfected with Ca_V_1.2 (pCa_V_1.2/pKK56/pMX125/pMX199)‐ or F) Ca_V_1.3 (pCa_V_1.3/pKK56/pMX125/pMX199)‐dependent CaB‐A systems were depolarized with 20 mm KCl and immediately seeded into culture wells containing different concentrations of CCBs. Data are mean ± SD of SEAP levels scored at 48 h after exposure to CCBs (n = 3 independent experiments).

PD drug discovery would greatly benefit from multiplexed drug screening, allowing simultaneous assessment of multiple disease‐specific drug targets within a single experiment^[^ ^14^ ^]^ (**Figure** **1**A). In cell‐based assay designs, secreted reporter proteins such as human placental secreted alkaline phosphatase (SEAP) or *Gaussia princeps* luciferase (GLuc) are advantageous for qualitative, non‐disruptive, and high‐throughput recording of gene expression, while on the other hand, intracellular reporter systems such as fluorescent proteins facilitate resource‐efficient and simple experimental setups.^[^ ^15^ ^]^ To design a CCB‐regulated reporter protein assay, we created a synthetic excitation‐transcription coupling system.^[^ ^16^ ^]^ Activation of Ca_V_1 channels by membrane depolarization triggers a surge in cytosolic [Ca^2+^]_i_, initiating different signal‐transduction pathways that modulate endogenous calcium‐specific promoters^[^ ^17^ ^]^ (Figure S1A, Supporting Information). Among different calcium‐specific promoters (CSP) known to respond to Ca_V_1‐dependent cell signaling,^[^ ^18^, ^19^, ^20^ ^]^ the synthetic NFAT promoter P_NFAT3_ (pMX57, P_NFAT3_‐SEAP‐pA; P_NFAT3_, (NFAT_IL4_)_5_‐P_min_) showed the most suitable Ca_V_1.2‐ and Ca_V_1.3‐dependent SEAP induction profiles triggered by potassium chloride (KCl)‐mediated depolarization (Figure S1B,S1C, Supporting Information). After validating dose‐dependent excitation‐transcription coupling with different secreted (Figure S2A,S2B, Supporting Information) and fluorescent reporter systems (Figure S2C, Supporting Information), we tested the potential of the cell‐based SEAP assay for CCB drug discovery. In a genetic configuration enabling CCB‐repressible reporter expression (CaB‐R assay) (Figure S3A, Supporting Information), the presence of CCBs blocking Ca_V_1.2 and Ca_V_1.3 inhibits NFAT signaling and causes a dose‐dependent decrease of SEAP production (Figure S3B,S3C, Supporting Information). When we validated CaB‐R with a selection of clinically approved CCB drugs, the IC_50_ values determined in this study generally lay within the reference ranges reported for both Ca_V_1‐channel isoforms (Table S1, Supporting Information). In addition, CaB‐R allowed for use‐dependent analysis of repetitive CCB‐mediated channel inhibition and activation, which is a critical but often elusive screening requirement in ion channel drug discovery.^[^ ^21^ ^]^ Following prolonged depolarization of cells loaded with Ca_V_1.2 or Ca_V_1.3‐dependent CaB‐R systems, the representative CCB nicardipine showed a typical use‐dependent channel antagonism profile characterized by stronger inhibition at hyperactive channel states (KCl = 20, 40 mm) as compared to the degree of inhibition at baseline channel activities (KCl = 0 mm) (Figure S3D,S3E, Supporting Information).^[^ ^22^, ^23^ ^]^

### 2.2 Engineering of an Antagonist‐Inducible Reporter Assay to Reduce False‐Positive Results

In many drug‐screening studies that involve the use of living cells, cytotoxicity‐mediated signal decrease often interferes with antagonist‐associated reporter signals, thus generating false‐positives.^[^ ^24^, ^25^ ^]^ To overcome this limitation, we engineered a CCB‐activated reporter assay (CaB‐A) that operates in a reversed configuration, allowing depolarization‐dependent NFAT signaling to repress reporter protein expression (Figure S4A, Supporting Information). The presence of CCBs antagonizes Ca_V_1‐mediated NFAT activation and triggers de‐repression of reporter gene transcription (Figure S4B, Supporting Information). Not only did CaB‐A reveal all CCB‐mediated drug effects in the expected dose‐dependent manner (Figure S4C,S4D, Supporting Information), but it also effectively reduced the risk of obtaining false‐positives, as expected. For example, cytotoxicity control experiments might have led to the classification of the CCB‐repressible effect of flunarizine as a false positive in CaB‐R (Figure S4E, Supporting Information), but the potency of flunarizine in activating gene expression in CaB‐A corroborated the true channel‐blocking efficacy of this drug (Figure S4C,S4D, Supporting Information). Baseline signal levels of CaB‐A assays could be further fine‐tuned by choosing different splice‐variants of each channel isoform (Figure S4F, Supporting Information), as we demonstrated with two alternatively spliced Ca_V_1.3 *α*1‐domains characterized by different basal channel activities^[^ ^10^ ^]^ (Figure S4G, Supporting Information).

In CaB‐A (Figure S4B, Supporting Information), CCB‐activated gene expression results from inhibition of NFAT‐repressible gene expression of a synthetic transcription factor, which binds to and silences synthetic cognate promoters driving constitutive expression of the reporter gene. However, most synthetic transcription factors—especially those having a TetR‐family repressor domain—are inherently under allosteric control by particular ligands.^[^ ^26^, ^27^ ^]^ Indeed, when we used a paraben‐dependent mammalian trans‐silencer (PMS, PmeR‐KRAB)^[^ ^28^ ^]^ as the NFAT‐driven repressor, we found that nicardipine and benidipine interfered with de‐repression of PMS‐specific promoters at high concentrations (>1 µm) (Figure S4H, Supporting Information), which would likely cause erroneous interpretation of the CaB‐A results (Figure S4C,S4D, Supporting Information). To improve screening accuracy, we designed an optimized CaB‐A configuration in which the synthetic NFAT promoter controls the expression of L7Ae (an archaeal ribosome‐derived RNA‐binding protein with high affinity for a C/D box‐aptamer motif)^[^ ^29^, ^30^ ^]^ (Figure 1B). The presence of CCBs prevents NFAT‐dependent L7Ae expression (pMX125, P_NFAT4_‐L7Ae‐pA; P_NFAT4,_ (NFAT_IL4_)_7‐_P_min_; Figure S5A, Supporting Information) and de‐represses translation of reporter mRNA engineered to contain cognate C/D‐box motifs in the 5’‐UTR (Figure 1B). Depolarization‐dependent production of L7Ae could knock down translation of SEAP mRNA harboring either one (pMX195, P_SV40_‐(C/D box)_1_‐SEAP‐pA) or two C/D‐box repeats (pMX199, P_SV40_‐(C/D box)_2_‐SEAP‐pA), with the vector combination of pMX125/pMX199 affording optimal nicardipine‐inducible SEAP expression characterized by low background signals and high induction profiles for use‐dependent Ca_V_1‐inhibition (Figure 1C,D). Importantly, this modified CaB‐A assay is no longer influenced by potential crosstalk between CCBs and the L7Ae‐C/D box interaction (Figure S5B–S5D, Supporting Information), and thus it enables accurate assessment of dose‐dependent CCB‐channel antagonism (Figure 1E,F).

### 2.3 Multiplexed and High‐Throughput Screen of Plant Essential Oils to Identify Ca_V_1.2 and Ca_V_1.3 Antagonism

**Figure 2:**
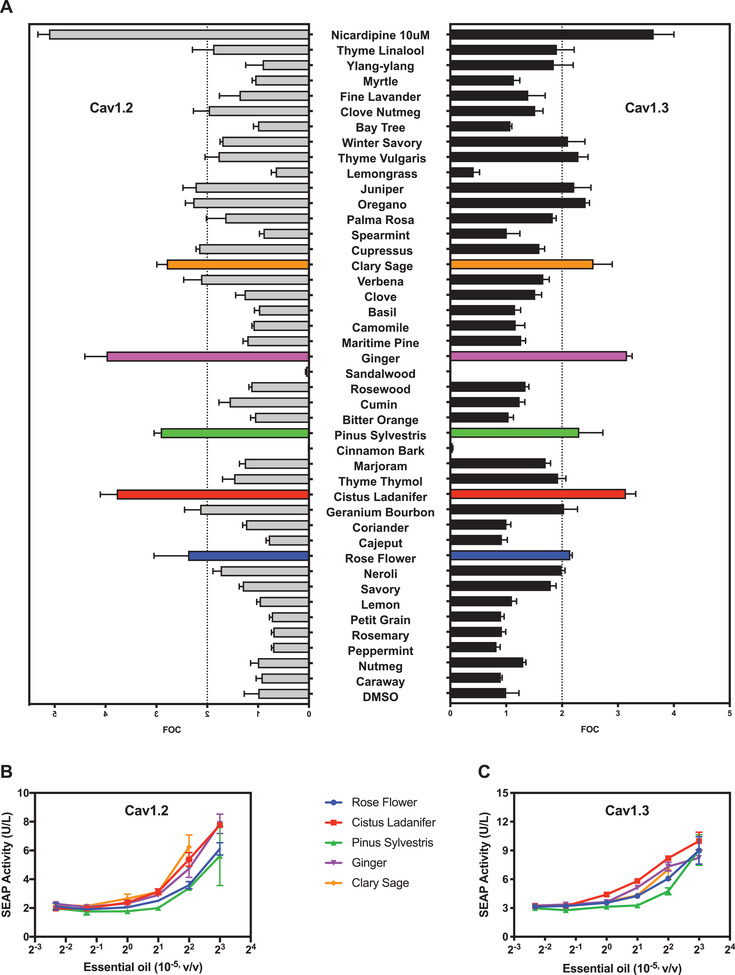
Identification of putative Ca_V_1.2‐ and Ca_V_1.3‐antagonizing essential oil products. A) High‐throughput analysis. Independent Ca_V_1.2‐ and Ca_V_1.3‐specific CaB‐A systems (pCa_V_/pKK56/pMX125/pMX199) were depolarized with 20 mm KCl and immediately seeded into culture wells supplemented with 4 × 10^−5^ v/v of different plant essential oils. Data are mean FOC (fold of DMSO control) ± SD of SEAP levels scored at 48 h after exposure to essential oils (n = 3 independent experiments). B,C) Dose‐dependent validation of the most active essential oil hits. (B) Ca_V_1.2‐ and (C) Ca_V_1.3‐specific CaB‐A systems were depolarized with 20 mm KCl and added to culture wells containing different essential oil dilutions (v/v). Data in (B,C) are mean ± SD of SEAP levels scored at 48 h after exposure to essential oils (n = 3 independent experiments). DMSO (solvent) levels in cell culture medium were kept below 0.4%.

Next, we used CaB‐A and performed a pilot test of HTS with a random selection of plant essential oils. Plant‐derived natural compounds have historically been proven to have great pharmacological potential,^[^ ^31^, ^32^ ^]^ especially for neurodegenerative diseases.^[^ ^33^ ^]^ In particular, plant‐derived compounds have inherently high “metabolite‐likeness” and bioavailability, and thus represent promising starting points for drug discovery.^[^ ^34^ ^]^ As plant‐derived natural products, essential oils can further be regarded as naturally selected packages of biocompatible, bioavailable, and bioactive substances.^[^ ^35^ ^]^ Among 42 essential oil products tested (Table S2, Supporting Information), the CaB‐A assay identified five oils (i.e., rose flower, cistrus ladanifer, pinus sylvestris, ginger, clary sage) that most effectively inhibited Ca_V_1.2 and Ca_V_1.3 (**Figure** **2**A). All five essential oils dose‐dependently activated SEAP expression in the CaB‐A assay (Figure 2B,C), and control experiments confirmed that none of these essential oils interfered with L7Ae activity or intracellular calcium signaling (Figure S6A, Supporting Information). Notably, the results obtained with clary sage (*Salvia sclarea*) essential oil corroborated the advantage of CaB‐A; although high concentrations of this oil were cytotoxic according to a reporter‐based assay determining protein production capacity (Figure S6B, Supporting Information), the unique antagonism‐inducible gene expression readout of CaB‐A (Figure 2B,C) ensured that the clary sage data was not excluded as false‐negative. In terms of assay quality, both CaB‐R and CaB‐A assays have excellent Z’ screening windows (Z’ factor (CaB‐R) = 0.68 ± 0.14; Z’ factor (CaB‐A) = 0.73 ± 0.07, n = 12 independent experiments), and therefore should be suitable for rapid, robust and resource‐efficient HTS. As already mentioned, treatment of PD requires a compound that can maximally inhibit Ca_V_1.3, but not Ca_V_1.2^7^. To quantify the antagonistic activities towards Ca_V_1.3 (PD drug target) and Ca_V_1.2 (PD anti‐target) simultaneously (i.e., in a multiplexed screening configuration; Figure 1A), we mixed individual cell populations of Ca_V_1.2‐specific CaB‐R and Ca_V_1.3‐specific CaB‐A systems, each driving a different reporter protein. This cell mixture was exposed to clinically approved CCB drugs (positive controls), negative control compounds (i.e., amitriptyline, tetracaine, lidocaine), and the five essential oil hits, in order to determine their impact on the depolarization‐dependent expression of SEAP (reflecting Ca_V_1.3 activity) and GLuc (reflecting Ca_V_1.2 activity) (Figure S6C, Supporting Information). The experimental results confirmed the multiplexed screening capability of our system. All five essential oils showed the required basic selectivity profile of maximal Ca_V_1.3 inhibition (highest CaB‐A score vs control) and minimal Ca_v_1.2 inhibition (closest CaB‐R score to the control).

### 2.4 Integration of In Silico Virtual Screening and Deep Learning Enables the Discovery of (6)‐Gingerol and Sclareol as Novel Ca_V_1.3‐Antagonists

**Figure 3:**
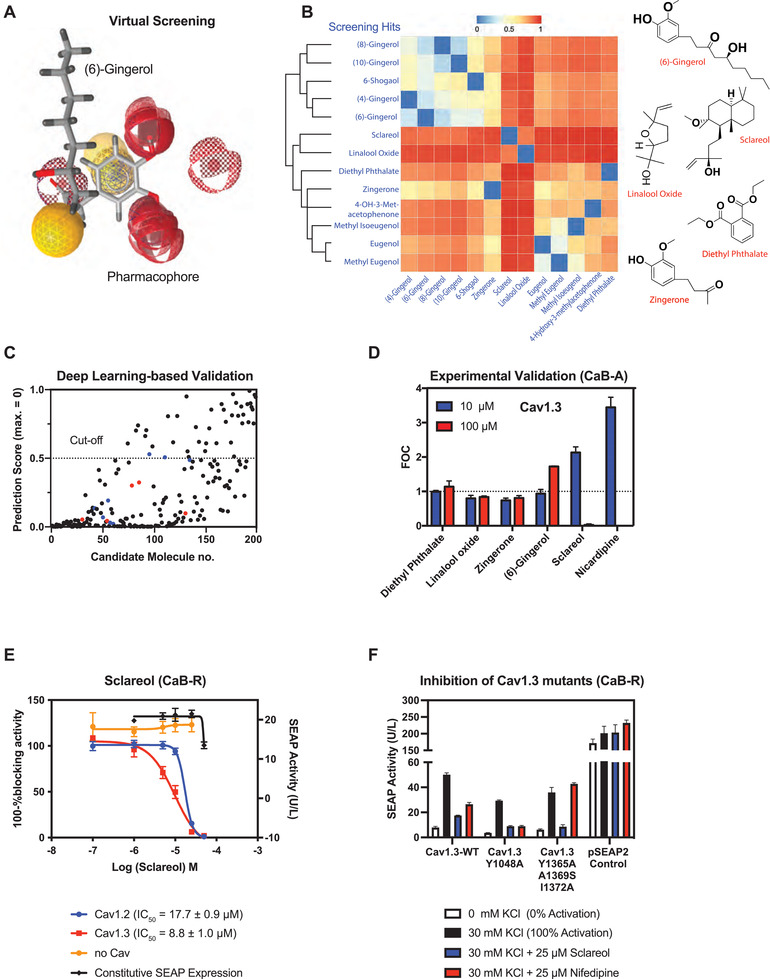
Identification of active constituents that selectively inhibit Ca_V_1.3. A) Ligand‐based virtual screening. Representative merged pharmacophore models for Ca_V_1.3 inhibitors created with LigandScout using the positive and negative reference compounds listed in Table S3, Supporting Information. This illustration exemplifies the alignment of (6)‐gingerol to the Ca_V_1.3‐blocking pharmacophores. B) Structure clustering analysis of candidate compounds. All 13 hits from the virtual screening experiment using 198 candidate molecules derived from GC‐MS data of essential oils (Table S3, Supporting Information) were clustered based on structure similarity using the ChemMine tool. Right panel: chemical structures of the five compounds selected as representatives of the clusters. C) Validation of virtual screening using a trained deep‐learning neural network. A D‐MPNN described in^[^ ^40^ ^]^ was trained with reported CCBs (Table S6, Supporting Information) as well as randomly chosen compounds from MUV datasets (47) in order to validate the 198 candidate molecules screened by LigandScout. Virtual screening hits in (B) are highlighted in blue (before clustering) and red (after clustering). Numerical values of rank‐ordered prediction scores (y‐axis) are listed in Table S5, Supporting Information. An arbitrary cut‐off of 0.5 was chosen to assess general goodness. D) Assessment of putative Ca_V_1.3 antagonism by the PD drug candidates. HEK‐293 cells transfected with the Ca_V_1.3‐dependent CaB‐A system were depolarized with 30 mm KCl and immediately seeded into culture wells containing different drug candidates supplemented at 10 or 100 µm. Data points are presented as mean FOC (fold of DMSO control) of SEAP levels scored at 48 h after exposure to nicardipine (n = 3 independent experiments). E) Quantification of Ca_V_1 antagonism by sclareol using CaB‐R. HEK‐293 cells transfected with Ca_V_1.2‐ or Ca_V_1.3‐dependent CaB‐R were depolarized with 30 mm KCl and immediately seeded into culture wells containing different concentrations of sclareol. HEK‐293 cells transfected with a constitutive SEAP‐expression vector (pSEAP2‐Control; P_SV40_‐SEAP‐pA) were used as a reference for putative cytotoxicity caused by drug exposure. HEK‐293 cells transfected with a bacterial expression vector (pViM41; P_T7_‐mCherry‐MCS) were used as a negative control indicating Ca_V_‐unrelated assay readouts. Data are mean ± SD of SEAP levels scored at 48 h after drug exposure (n = 3 independent experiments). F) Quantification of Ca_V_1 antagonism by sclareol and nifedipine on different Ca_V_1.3 mutants. HEK‐293 cells transfected with CaB‐R regulated by different synthetic Ca_V_1.3 mutants (WT, pCa_V_1.3/pKK56/pMX57; Ca_V_1.3^Y1048A^, pWH154/pKK56/pMX57; Ca_V_1.3^Y1365A, A1369S, I1372A^, pWH155/pKK56/pMX57) were depolarized with 30 mm KCl and immediately seeded into culture wells containing different concentrations of sclareol or nifedipine. HEK‐293 cells transfected with a constitutive SEAP‐expression vector (pSEAP2‐Control; P_SV40_‐SEAP‐pA) were used as a reference for putative cytotoxicity caused by drug exposure. Data are mean ± SD of SEAP levels scored at 48 h after drug exposure (n = 3 independent experiments).

To identify the putative active constituents of the five essential oils accounting for inhibition of Ca_V_1.3, we used the LigandScout software to perform ligand‐based virtual screening.^[^ ^36^, ^37^ ^]^ First, we generated 3D pharmacophore models of putative Ca_V_1.3 blockers (**Figure** **3**A) based on a series of Ca_V_1 blockers^[^ ^38^ ^]^ (positive reference), as well as Ca_V_‐independent ion channel modulators found in the multiplexed screening experiment (negative reference) (Figure S6C and Table S3, Supporting Information). Using these pharmacophore models, we performed in silico analysis of all constituents of rose flower, cistus ladanifer, pinus sylvestris, ginger, and clary sage essential oils by computing the similarity of each chemical structure to a theoretically ideal pharmacologically active moiety. From a total of 198 different candidate molecules, this virtual screening experiment generated 13 hits as the most promising Ca_V_1.3‐antagonists (Table S4, Supporting Information), and structure clustering analysis enabled us to select the five chemicals diethyl phthalate, linalool oxide, zingerone, (6)‐gingerol and sclareol as representative structures (Figure 3B). Parallel artificial intelligence (AI)‐based validation experiments based on a directed‐message passing neural network (D‐MPNN)^[^ ^39^, ^40^ ^]^ gave similar results (Figure 3C; Table S5, Supporting Information), achieving a receiver operating characteristic curve‐area under the curve (ROC‐AUC) value of 97.78%. Experimental testing of these 5 candidate compounds with the CaB‐A assay confirmed that (6)‐gingerol and sclareol showed Ca_V_1.3‐antagonistic activity (Figure 3D). Notably, both compounds showed a stronger inhibitory effect on Ca_V_1.3‐mediated reporter gene expression than on the Ca_V_1.2‐dependent CaB system (Figure 3E; Figure S6D–S6F, Supporting Information), and were also predicted to have optimal drug‐likeness properties according to Lipinski's Rule of Five criteria.^[^ ^41^ ^]^ Importantly, sclareol (8.8 ± 1.0 µm; Figure 3E) had a more than threefold lower IC_50_ value for Ca_V_1.3 than (6)‐gingerol (30.5 ± 6.3 µm; Figure S6E, Supporting Information) and is also structurally divergent from all currently known CCB compounds (Table S3, Supporting Information), such as dihydropyridines (DHP), represented by nifedipine (Figure 3B). Indeed, when we created putative DHP‐insensitive Ca_V_1.3 mutants based on amino acid alterations that were previously shown to be critical for CCB‐sensitivity of the related L‐type Ca_V_1.1 channel,^[^ ^42^, ^43^ ^]^ we found that our synthetic Ca_V_1.3^Y1365A, A1369S, I1372A^ mutant was no longer inhibited by nifedipine, but still retained full sensitivity to sclareol (Figure 3F). This result suggests that the binding modes of sclareol and DHPs to Ca_V_1.3 are different. These features render sclareol a promising lead compound for PD pharmacotherapy.

### 2.5 Validation of Neuroprotective Activity of Sclareol In Vitro and in Mice

To assess the potential in vivo efficacy of sclareol, we first confirmed the presence of its molecular target in SNc DA neurons by immunostaining of midbrain‐containing brain slices for Ca_V_1.3 and tyrosine hydroxylase (TH), which are known to be co‐expressed in this brain area^[^ ^44^ ^]^ (Figure S7A, Supporting Information). To demonstrate functional Ca_V_1.3 inhibition by sclareol, we next performed whole‐cell patch‐clamp recordings of SNc DA neurons. Bath‐application of 10 µm sclareol led to a significant neuronal hyperpolarization (−62 ± 2 mV vs −80 ± 2 mV, *p* = 0.0001). This was accompanied with an increased spiking threshold (rheobase 3.75 ± 0.6 pA vs 11 ± 1.8 pA, *p* = 0.0107) and decreased firing responses to incremental current injection steps (two‐way repeated model ANOVA, sclareol effect F(1,12) = 19.49, *p* = 0.0008, Figure S7B, Supporting Information). These in vitro findings confirm that sclareol significantly decreases the excitability of SNc DA neurons.

**Figure 4:**
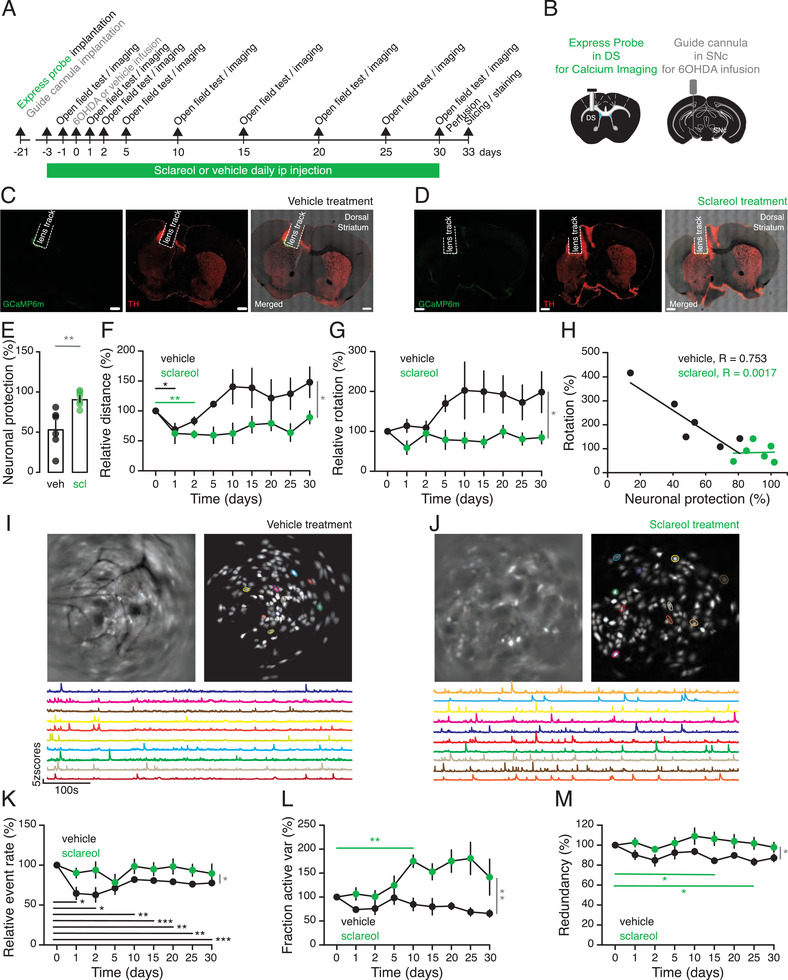
Sclareol exhibits in vivo neuroprotective effects against 6‐OHDA‐induced degeneration. A) Experimental set‐up for simultaneous monitoring of locomotion behavior and live neuronal dynamics imaging in 6‐OHDA‐infused PD mice treated with daily doses of either sclareol (55 mg kg^−1^) or vehicle as a negative pharmacologic control. B) Schematic illustration of the surgical placement of the express probe and the miniature microscope above the DS for live imaging of calcium dynamics of striatal neurons as well as the guide cannula above the ipsiversive SNc to infuse the PD‐triggering neurotoxin 6‐OHDA. C,D) Representative confocal micrographs of a DS section of a vehicle‐treated control (C) and sclareol‐treated (D) animal showing the track left behind the lens and expression of GCaMp6m immediately below. The tissue was stained for DA neuron‐specific marker TH (red). E) Sclareol‐mediated neuronal protection level quantified by differential TH staining with the contraversive hemispheres in sclareol and vehicle treatment groups. F) Relative distance traveled over time by the sclareol and vehicle treatment groups. G) Relative contraversive rotations performed by both mouse groups at each time point of the experiments. H) Correlation between the relative number of contraversive rotations and the neuronal protection levels of sclareol and vehicle treatment groups. I,J) Representative raw live calcium imaging and profiling of individual cells (circled in different colors) and the corresponding dynamic calcium time courses (matching colors) recorded from vehicle‐treated (I) and sclareol‐treated (J) animals using the head‐mounted miniaturized microscope. K) Relative calcium transients event rates for sclareol and vehicle treatment groups. L) Relative variance of the active neuronal fraction over time for sclareol and vehicle treatment groups. M) Relative neuronal redundancy time course for sclareol and vehicle treatment groups. Data are relative to the values obtained on day 0 before the infusion of 6‐OHDA (n = 6; scale bar 500 µm). Data are shown as the mean ± SEM, statistics by unpaired *t*‐test (E) or two‐way repeated‐measures ANOVA test (F, G, K–M), n = 6 mice per group. *p<0.05, **p<0.01, ***p<0.001, stats shown in black and green relate to vehicle‐ and sclareol‐ treated mice respectively, whereas stats shown in grey relate to the comparison between both groups.

**Figure 5:**
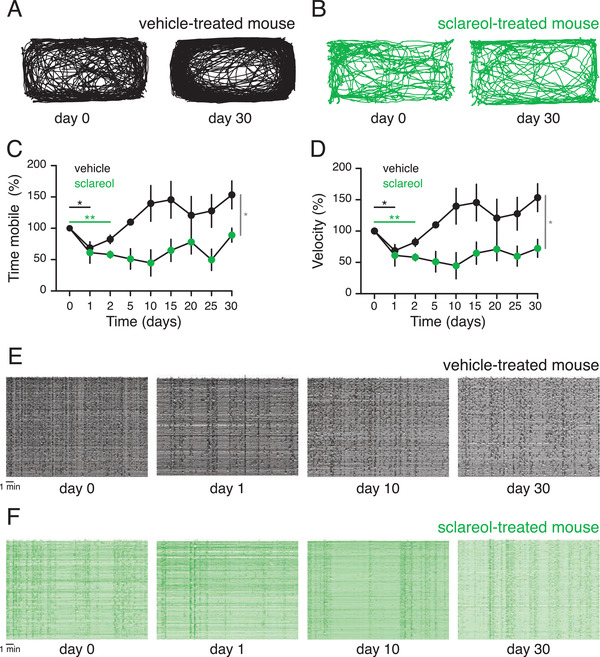
Sclareol prevents locomotion deficits and over‐synchronization of striatal neurons. A) Representative tracking trace of the vehicle‐treated mouse at baseline (day 0) and at the end of the procedure (day 30). B) Same as in (A) but from a sclareol‐treated mouse. C) Relative mobility time throughout the experiment for both mouse groups. D) Relative movement velocity over time for both mouse groups. E) Z score of the calcium traces of striatal neurons from the vehicle‐treated mouse showing the neuronal activity redundancy at days 0, 1, 10, and 30. F) Same as in (E) but from striatal cells of a sclareol‐treated mouse. Data are shown as the mean ± SEM, statistics by two‐way repeated‐measures ANOVA test (C, D), n = 6 mice per group. *p<0.05, **p<0.01.

To confirm sclareol's neuroprotective effect in experimental PD, we concomitantly profiled the locomotion behavior and neuronal dynamics of live sclareol‐treated versus vehicle‐treated PD model mice^[^ ^45^ ^]^ (**Figure** **4**, **Figure** **5**, Movies S1 and S2, Supporting Information). An express probe consisting of a GRIN‐lens coated with the genetically‐encoded calcium sensor AAV‐CaMKII‐GCaMP6m was first implanted above the dorsal striatum (DS) to enable real‐time monitoring of calcium dynamics from striatal neurons, along with a guide cannula above the ipsiversive SNc to infuse the PD‐triggering neurotoxin 6‐hydroxydopamine (6‐OHDA) (Figure 4A–C). Three weeks after the surgery, a miniaturized microscope was mounted on the animals’ heads to image calcium‐associated striatal neuron dynamics in real‐time as the animals explored an open arena, thereby setting the baselines for behavioral and neuronal activities. The animals were treated with daily doses of either sclareol (55 mg kg^−1^) or vehicle as a negative pharmacologic control, starting two days before and throughout the 30 days post the single 6‐OHDA infusion (Figure 4A,B). The extent of PD‐associated neuronal degeneration was confirmed by immunostaining of the DA neuron‐specific marker TH, which was found to be significantly decreased in the ipsiversive DS compared to the contralateral control side of the vehicle control group (Figure 4C,E, 54 ± 10%). In contrast, in the sclareol‐treated mouse group little‐to‐no loss in DA neurons was observed when compared to the contraversive control side as well as to the vehicle‐treated‐ mouse group (Figure 4D,E, 92 ± 4%; *p* = 0.0061 sclareol‐ vs vehicle‐ control group).

When allowed to explore an open arena to monitor locomotion performance, vehicle‐treated PD mice carrying the head‐mounted mini‐endoscopic microscope and a cannula developed strong locomotion alterations from baseline throughout the entire experimental timespan, while sclareol‐treated animals showed consistently stable travel distance, mobility time, and velocity and appeared to be unaffected by PD (Figure 4F and Figure 5A–D). Consistently, vehicle‐treated PD mice also manifested locomotion impairments, exhibiting unilateral rotations contraversive to the 6‐OHDA‐affected hemisphere (Figure 4G and Movie S1, Supporting Information). Interestingly, sclareol‐treated mice showed neither 6‐OHDA‐triggered locomotion increase nor compensatory rotational behavior (Figure 4F,G; Figure 5A–D; Movie S2, Supporting Information). Additionally, there was an inverse correlation between rotational behavior and neuronal integrity, suggesting that sclareol was indeed preserving the locomotion capabilities of treated animals and protecting them from PD‐associated deficiencies (Figure 4H).

Monitoring of striatal neuron activities recorded in real time using mini‐endoscopic live single‐cell calcium imaging showed a significant difference in the calcium dynamics of sclareol‐treated mice, compared with PD mice (Figure 4I–M). Decreased calcium dynamics is known to correlate with a reduced event rate of striatal neurons due to a PD‐associated loss of DA neuromodulation.^[^ ^46^ ^]^ Here, the calcium transients event rate (Figure 4K, two‐way repeated model ANOVA, F(8,40) = 4.64, *p* <0.0001) was indeed reduced in vehicle‐treated mice. And most importantly the relative calcium event rate (Figure 4K, two‐way repeated model ANOVA, sclareol effect F(10,80) = 6.90, *p* <0.0001), the relative variance of the active neuronal fraction (Figure 4L, two‐way repeated model ANOVA, sclareol effect F(10,80) = 11.08, *p* <0.0001) and the neuronal redundancy over time (Figure 4M, two‐way repeated model ANOVA, sclareol effect F(10,80) = 9.50, *p* <0.0001, and Figure 5E,F) were all significantly different in PD mice compared to sclareol‐treated animals. Thus, the calcium live‐imaging recordings confirm that striatal neurons in these mouse groups encode motion in a distinct way. Overall, these data support the idea that sclareol protects the animals against the development of PD‐associated deficiencies in locomotion programmed by DS neurons.

## 3 Discussion

Synthetic biology is currently undergoing a transition from a design‐driven era of creating template circuits into a demand‐driven discipline focused on the creation of problem‐solving cell functions.^[^ ^47^, ^48^, ^49^ ^]^ By engineering synthetic gene circuits customized to quantify Ca_V_1.2 and Ca_V_1.3 activities individually, we were able to overcome a major technical obstacle to drug discovery for PD. When compared to the FLIPR assay (fluorescence imaging plate reader), which is considered the current gold‐standard for HTS of ion channel modulators, our cell‐based CaB‐R and CaB‐A assays offer numerous advantages: i) high Z’‐scores, which are pivotal for HTS of large sample volumes, ii) compatibility with use‐dependent analysis of CCBs to increase the information content of individual screens,^[^ ^21^, ^50^ ^]^ and iii) multiplexed target analysis enabling one‐step assessment of massively parallel drug targets and anti‐targets under identical screening conditions.^[^ ^24^, ^51^ ^]^ By using CaB‐R and CaB‐A in combination with computer‐aided technologies, such as virtual screening and deep learning, we were able to identify sclareol as a novel drug candidate for neuroprotection in PD patients. In terms of in silico drug discovery, we used LigandScout for the screening of novel drug candidates as it uses an effective algorithm to rationally compute pharmacophores based on molecular structures of chemical compounds with known drug properties.^[^ ^37^ ^]^ Deep‐learning‐based approaches can also be used for this purpose,^[^ ^39^ ^]^ but we only trained our D‐MPNN with known CCBs (Table S6, Supporting Information) without further optimizing the model through iterative cycles between experimental validation and additional training. Nevertheless, it showed excellent utility for the validation of our LigandScout results. Interestingly, both LigandScout and D‐MPNN predicted stronger channel antagonism for linalool oxide and gingerol versus sclareol, but sclareol prevailed in subsequent experimental validations. This suggests that cell‐based assays may provide a more advanced proxy than in silico technology for drug discovery.

Sclareol is a natural compound derived from the Mediterranean medicinal plant *Salvia sclarea* (clary sage), has selectivity for Ca_V_1.3 over Ca_V_1.2, waspreviously demonstrated to attenuate growthand cell cycle progression of human leukemic cells,^[^ ^52^ ^]^ shows low systemic toxicity and good bioavailability in vivo, and can penetrate the blood‐brain barrier following oral intake. In addition, sclareol is structurally divergent from all L‐type voltage‐gated CCBs known to date, and might therefore have a unique pharmacological profile without the common limitations of currently available PD drugs. All these features are favorable for potential clinical application. In a mouse model of PD, we could indeed confirm promising therapeutic effects of sclareol, including prevention of SNc DA neuronal degeneration and maintenance of motor performances as compared to control PD mice. Notably, as PD patients typically show decreases in locomotion, the abnormally high locomotion parameters of our vehicle‐treated PD mice may seem counter‐intuitive. However, this observation can be readily explained in terms of the constraints of our experimental model, since the 6‐OHDA infusions were done unilaterally and not bilaterally. Such unilateral infusions trigger an unbalanced motor command between hemispheres, leading to unilateral rotations contraversive to the PD‐affected hemisphere. This phenomenon is widely known, and similar findings of contraversive rotational behavior were reported in unilaterally 6‐OHDA‐infused rodents treated with L‐DOPA.^[^ ^53^ ^]^

Collectively, we believe this work well illustrates the value of multi‐faceted experimental and computational drug discovery platforms, and especially the utility of cell‐based solutions created with synthetic biology‐inspired engineering principles, which we employed here to tailor the first high‐throughput multiplexed drug screening system for ion channel‐related diseases. This platform enabled us to identify sclareol as a structurally distinctive lead compound/candidate drug for neuroprotection in PD patients. We anticipate that the combination of molecular medicine, high‐throughput technologies, and AI exemplified in this work will have a huge impact in many areas of biomedicine.

## 4 Experimental Section

### Vector Design

Comprehensive design and construction details for all expression plasmids are provided in Table S7, Supporting Information.

### Computer‐Aided Drug Screening

Representative pharmacophore models were created with LigandScout software^[^ ^37^ ^]^ based on the reference ion channel blockers listed in Table S3, Supporting Information. To perform alignments to the pharmacophore, all constituents of rose flower, cistus ladanifer, pinus sylvestris, ginger, and clary sage obtained from GC‐MS data kindly provided by Welfine Beijing Science & Technology Development Co. Ltd (Beijing, China) were assigned with a chemical SMILES (Simplified Molecular‐Input Line‐Entry System) language. Suggested hits of virtual screening were refined by structure similarity analysis using the free ChemMine software (http://chemmine.ucr.edu/).^[^ ^54^ ^]^ To train a D‐MPNN,^[^ ^40^ ^]^ isomeric SMILES strings of published CCBs (Table S6, Supporting Information; positive reference, labeled 0) and 400 random compounds from maximum unbiased validation (MUV) datasets^[^ ^55^ ^]^ (negative reference, labeled 1) were used. After training, the binary classification model was applied to predict the goodness of candidate molecules from the same GC‐MS dataset used for virtual screening (309 constituents from five essential oils, 198 non‐redundant chemical compounds). Rank‐ordered prediction scores (y‐axis) are listed in Table S4, Supporting Information. Drug‐likeness properties of candidate Ca_V_‐blocker drugs, including pharmacokinetics, Lipinski's Rule of Five criteria,^[^ ^41^ ^]^ and blood‐brain barrier permeability, were evaluated using the ADME toxicity predictor SwissADME (https://www.click2drug.org/directory_ADMET.html).

### Whole‐Cell Patch Clamp Recordings

Acute coronal slices (200 µm) containing SNc were prepared using a vibrotome (Leica) in ice‐cold cutting solution (in mm: NMDG 92, KCl 2.5, NaHPO_4_ 1.25, NaHCO_3_ 30, HEPES 20, glucose 25, thiourea 2, Na‐ascorbate 5, Na‐pyruvate 3, MgSO_4_.7H_2_O 10, CaCl_2_.4H_2_O 0.5, and N‐acetylcysteine 10, pH 7.3, 290–300 mOsm). Slices were incubated in ACSF solution (in mm: NaCl 92, KCl 2.5, NaH_2_PO_4_ 1.25, NaHCO_3_ 30, HEPES 20, glucose 25, thiourea 2, Na‐ascorbate 5, Na‐pyruvate 3, MgSO_4_.7H_2_O 2, CaCl_2_.4H_2_0 2, pH 7.3, 290–300 mOsm) at 31 °C. Slices were then transferred to the recording chamber, superfused with Ringer solution (in mm: NaCl 119, KCl 2.5, NaH_2_PO_4_ 1.25, NaHCO_3_ 24, glucose 12.5, CaCl_2_.4H_2_O 2 and MgSO_4_.7H_2_O, pH 7.3, 290–300 mOsm) at 2 mL min^−1^ under bubbling with 95% O_2_ and 5% CO_2_. Neurons were visualized with an IR camera on an Olympus scope U‐TV1X‐2 and whole‐cell patch‐clamp recordings (multiclamp 700B amplifier) were performed. The internal solution for voltage clamp recordings contained (in mm): K‐gluconate 130, creatine phosphate 10, MgCl_2_ 4, Na_2_ATP 3.4, Na_3_GTP 0.1, EGTA 1.1 and HEPES 5, pH 7.3, 289 mOsm. Cells were clamped at −70 mV.

### 6‐OHDA‐Induced Mouse Model of Parkinson's Disease

WT mice (male of 6 to 8 weeks old) were ip injected daily with either sclareol (55 mg kg^−1^) or vehicle (5% EtOH, 5% Cremophor; v/v in ddH_2_O). 6‐OHDA was infused via a custom guide cannula (460 µm in diameter and 6.1 mm in length, P1 Technologies) placed unilaterally above the SNc (AP: −3.4; L: −1.5, and DV: −4.0 mm). The cannula was filled with a dummy cannula of the same length and covered by temporary silicone gel (KauPo). Two days after the first sclareol injection, mice were infused with 500 nL of 5 mg mL^−1^ 6‐OHDA solution in 0.9% (w/v) NaCl with 0.02% (w/v) ascorbic acid (flow rate of 0.1 µL min^−1^). Histological procedures were performed 30 days post‐6‐OHDA infusion.

### Immunohistochemistry

Mice were anesthetized with a lethal i.p. injection of pentobarbital (300 mg kg^−1^) and perfused intracardially with cold PBS followed by 4% paraformaldehyde. The brains were extracted and kept in 4% sucrose until complete saturation. Slices (60 µm thick) containing the DS were cut with a cryostat and processed for TH and Ca_v_1.3 immunohistochemistry. Slices were washed for 3 min 3 times in TBS (1X), permeabilized with TBS containing 0.1% Tween 20 and 0.1% Triton X100, washed again for 3 min 3 times in TBS (1X), and blocked in 1% BSA‐TBS solution for 2 h. Finally, mouse anti‐TH antibody and/or anti‐CaV1.3 (1/500, Sigma) was added and slices were softly shaken at 4 °C overnight. Next day, the slices were washed for 3 min 3 times in TBS (1X) and incubated with Alexa 488 donkey anti‐rabbit antibody or Alexa 555 donkey anti‐mouse antibody (1/500, Sigma) for 2 h at room temperature. After a last round of TBS washing, the slices were mounted on slides and imaged with a confocal microscope (Zeiss LSM700). Images were processed with ImageJ and the fluorescence intensity was analyzed with Matlab (Mathwork).

### In Vivo Single‐Cell Calcium Imaging

An express probe (carrying the AAV1.Camk2a.GCaMP6m.WPRE.SV40, Ready to Image virus, Inscopix) was unilaterally placed above the ipsiversive DS to the cannula (AP: +0.6; L: −1.7; and DV: −2 mm). The probe was fixed with a UV‐light‐curable glue (Henkel). A custom‐made head bar (2 cm long, 0.4 cm wide, 0.1 cm tall) was placed for future handling. A fixed headcap was built from layers consisting of super‐glue (Cyberbond), UV‐light‐curable glue (Loctite), and dental cement (Lang). Small screws were anchored in the skull to improve adhesion between the skull and the head cap. The headcap was secured to the skin with Vetbond tissue adhesive glue (3m). The expression of GCaMP6m and the clearing of the lens were assessed regularly starting from 10 days post‐surgery. Calcium transients were recorded with the nVoke2 system, pre‐processed in the Inscopix Data Processing Software (IDPS, v1.3, Inscopix), and finally processed and analyzed with Python in collaboration with Inscopix.

### Ethics

Male and female C57BL/6JRj mice were bred in‐house. No gender differences were observed, and the data were thus pooled. All experimental procedures were approved by the Institutional Animal Care Office of the University of Basel and the Cantonal Veterinary Office under License Number 2742.

### Statistical Analysis

CCB‐activity in CaB‐R assays was calculated as “percentage of control”, with reporter protein levels normalized to maximum average counts (100%; 40 mm KCl addition) and minimum average counts (0%; 10 µm nicardipine). Normalization calculations and nonlinear regression curve‐fittings (log (inhibitor) normalized response–variable slope), and statistical analysis were all conducted in Prism 7.0 (Graph Pad Software, San Diego, CA, USA). For statistical analysis, an extra sum‐of‐squares F test was performed to determine the significance of differences in Log(IC_50_) among the data sets of Figure S3D,E, Supporting Information. Fold of control (FOC) values were calculated as FOC = X*i*/avg(c_+_) × 100, where X*i* is the measurement of the i^th^ compound and avg(c_+_) is the average measurement of the DMSO‐treated samples. The Z’ value was calculated between the positive (10 µm nicardipine) and negative (0.1% DMSO) controls according to the reported equation (Zhang et al., 1999). All values for in vitro experiments are expressed as the mean ± SD.

Whole‐cell patch‐clamp results (Figure S7B, Supporting Information) were analyzed by paired *t*‐test or two‐way repeated‐measures ANOVA. Immunohistochemistry (Figure 4E) results were analyzed by un‐paired *t*‐test. Mouse behavior and in vivo single‐cell calcium imaging results were analyzed by two‐way repeated‐measures ANOVA. All the above data sets for sclareol efficacy tests in vivo are shown as mean ± SEM. *p<0.05, **p<0.01, ***p<0.001.

## Conflict of Interest

The authors declare no conflict of interest.

## Author Contributions

H.W. conceived the project. H.W., M.X., G.R., and K.T. designed the experiments. H.W., G.R., and K.T performed the experimental work in vitro and in mice. H.W. and X.L. conducted all in silico experiments. H.W., M.X., K.T., and M.F. analyzed the results. H.W., M.X., K.T., and M.F. wrote the manuscript.

## Supporting information

Supporting Information

Supporting Movie 1

Supporting Movie 2

## Data Availability

The data that support the findings of this study are available from the corresponding author upon reasonable request.
