# Supplementary material for: Identification of Sclareol As a Natural Neuroprotective Cav1.3‐Antagonist Using Synthetic Parkinson‐Mimetic Gene Circuits and Computer‐Aided Drug Discovery
Source: Adv Sci (Weinh). 2022 Jan 18;9(7):2102855. doi: 10.1002/advs.202102855 (PMC8895113; doi:10.1002/advs.202102855)
Supplement: Supplementary file 1 — Supporting Information [file ADVS-9-2102855-s003.pdf]

ADVANCED SCIENCE
Open Access

Supporting Information

for *Adv. Sci.*, DOI: 10.1002/advs.202102855

Identification of Sclareol As a Natural Neuroprotective $Ca_v1.3$-Antagonist Using Synthetic Parkinson-Mimetic Gene Circuits and Computer-Aided Drug Discovery

Hui Wang, Mingqi Xie, Giorgio Rizzi, Xin Li, Kelly Tan, and Martin Fussenegger**

Supporting Information

Identification of sclareol as a natural neuroprotective Cav1.3-antagonist using synthetic Parkinson-mimetic gene circuits and computer-aided drug discovery

Hui Wang[1], Mingqi Xie[1], Giorgio Rizzi[2], Xin Li[1], Kelly Tan[2,] and Martin Fussenegger[1,3]**

Methods

Vector Design. Mouse $Ca_V1.2$ α1C (GenBank accession number AY728090, Addgene plasmid #26572), rat $Ca_V1.3$α1D (GenBank accession number: AF370009, Addgene plasmid # 49333), rat $Ca_V1.3$α1D:Δ42a (GenBank accession number: AF370010, Addgene plasmid # 49332), rat Ca_Vβ3 (GenBank accession number: M88751, Addgene plasmid # 26574) and rat Ca_Vα2δ-1 (GenBank accession number: AF286488, Addgene plasmid # 26575) were provided by Prof. Diane Lipscombe (Brown University). ClonExpress® MultiS One Step Cloning Kit (cat. no. C113-01) was purchased from Vazyme Biotech Co. Ltd. (Nanjing, China).

Cell culture and transfection. Human embryonic kidney cells (HEK-293T, ATCC: CRL-11268) were cultured in Dulbecco's modified Eagle's medium (DMEM; Invitrogen, Basel, Switzerland; cat. no. 52100–39) supplemented with 10% (v/v) fetal bovine serum (FBS; Sigma-Aldrich, Buchs, Switzerland; cat. no. F7524, lot no. 022M3395) and 1% (v/v) penicillin/streptomycin solution (PenStrep; Biowest, Nuaillé, France; cat. no. L0022-100). Cells were cultured at 37°C in a humidified atmosphere of 5% CO_2 in air. For passaging, cells of pre-confluent cultures were detached by incubation in 0.05% trypsin-EDTA (Life Technologies, CA, USA; cat. no. 25300-054) for 3 min at 37°C, collected in 10 ml of cell culture medium, centrifuged for 3 min at 290 g, and resuspended in fresh culture medium at 1.5 x 10^5 cells/mL, before seeding into new tissue culture plates. Cell number and viability were quantified using an electric field multichannel cell counting device (Casy Cell Counter and Analyzer Model TT, Roche Diagnostics GmbH). HEK-293T cells were transfected using a polyethyleneimine (PEI)-based protocol. In brief, 2 x 10^5 cells/ml cells were seeded into 48-well plates (225 µl medium per well), 6-well plates (1800 µl medium per well), or 10 cm petri dishes (9 mL medium). At 24 h after seeding, cells were incubated for 12 h with 50 µl, 100 µl or 400 µl of a 10:3 PEI:DNA mixture (w/w), containing 0.3-0.4 µg (48-well plate), 2.4-3 µg (6-well plate), or 11-13 µg of total DNA (10 cm petri dish), respectively. For

experiments performed in 24-well plates, the culture medium was exchanged to PEI-free medium containing defined concentrations of control compounds after 12 h. For experiments performed in 6-well plates or 10-cm dishes, transfected cells were detached with 0.05% trypsin-EDTA, re-seeded into 96-well plates (15000-40000 cells/well), and cultured in medium containing defined concentrations of control compounds. Analytical reporter assays were performed at 48 h after medium exchange.

Chemicals and drugs. Ethanol (EtOH; cat. no. 02860), sclareol (cat. no. 357995), linalool oxide (cat. no. 62141), zingerone (cat. no. 88787), Cremophor (cat. no. C5135) and dimethyl sulfoxide (DMSO; cat. no. D8418) were purchased from Sigma-Aldrich (Buchs, Switzerland). Polyethyleneimine (PEI; cat. no. 24765-2; stock solution 1 mg/ml in ddH_2O) was purchased from Polysciences (Eppelheim, Germany). (6)-Gingerol (cat. no. sc-201519) and diethyl phthalate (cat. no. sc-239738) were purchased from Santa Cruz Biotechnology (Heidelberg, Germany). Potassium chloride (KCl; cat. no. A3582; stock solution 4 M in ddH_2O) was purchased from AppliChem (Darmstadt, Germany). Calcium channel blocker (CCB) drugs (**Table S8**) and plant essential oils (**Table S2**) were stored in DMSO and diluted with DMEM to final working concentrations. Final DMSO levels in the cell culture medium were kept below 0.4%.

Quantification of target gene expression. Expression levels of human placental secreted alkaline phosphatase (SEAP) in culture supernatants were quantified based on p-nitrophenyl phosphate-derived light absorbance at 415 nm[28]. Gaussia Luciferase (GLuc) levels in culture supernatants were profiled using the BioLux® Gaussia luciferase assay kit (New England Biolabs, Ipswich, MA; cat. no. E3300). Fluorescence levels of HEK-293T cells grown in a black 96-well plate with a transparent bottom (Greiner Bio-one; cat. no. 655090) and cultivated in phenol red-free DMEM (ThermoFisher Scientific, cat. no. 21063029) were quantified with a microplate reader (Infinite M1000 PRO plate reader, Tecan Group Ltd). TurboGFP levels were recorded at excitation 460/9 nm, emission 502/20 nm. dsRed-Express levels were recorded at excitation 554/9 nm, emission 586/20 nm.

Fluorescence imaging. Fluorescence microscopy was performed with an inverted fluorescence microscope (Nikon Ti-E; Nikon) equipped with an incubation chamber, an Orca Flash-4 digital camera (Hamamatsu), a pE-100-LED (CoolLED) as the transmission light source, a Spectra X (Lumencor) as the fluorescent light source and a 4× objective, an excitation and emission filter set (TurboGFP: 475/525 nm; dsRed-Express 549/593 nm) and NIS Elements AR software (version 4.3.0).

Locomotion. Mice were placed in the center of a rectangular open arena (30 cm in width and 45 cm in length, with 30 cm tall walls) for 10 minutes. The miniscope was placed and secured on the head, then the mouse was translocated into the open arena, where its position was tracked and recorded on a downward-facing camera with ANYMAZE.

Supplementary Figures

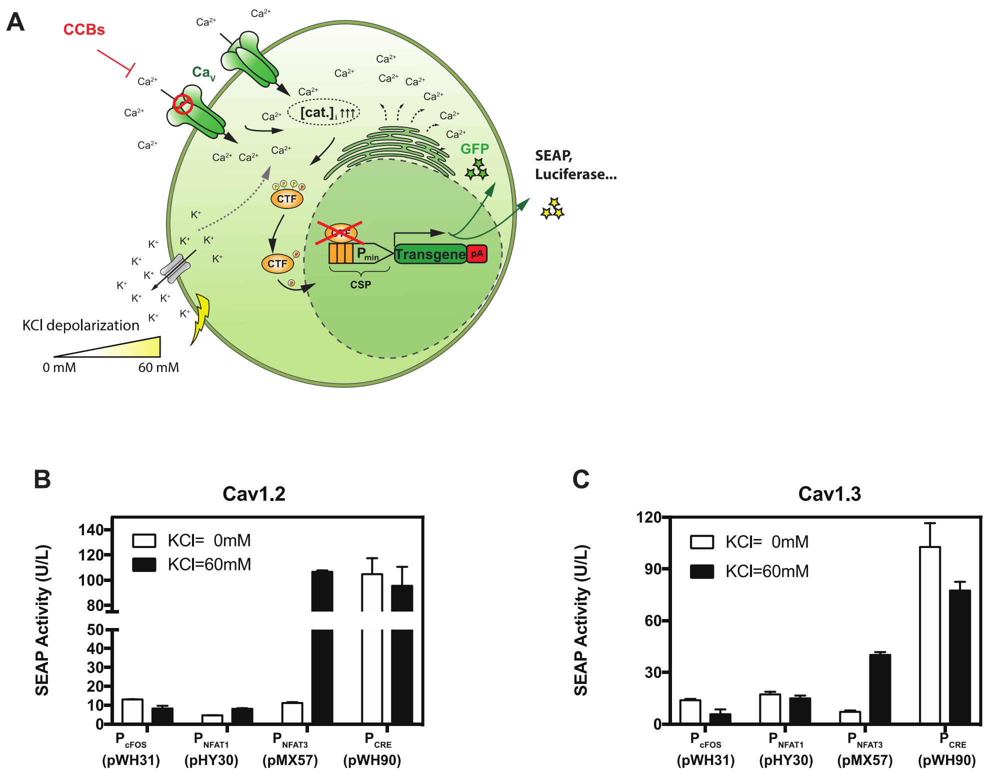

Figure S1. Principles of Ca_V1-specific designer cell-based screening assays. (A) Synthetic excitation-transcription coupling. In human embryonic kidney (HEK-293) cells, membrane depolarization activates L-type voltage-gated calcium channels (Ca_V1) and triggers Ca^{2+} influx, activation of endogenous calcium-responsive transcriptional factors (CTFs) and initiation of reporter gene transcription from synthetic cognate calcium-specific promoters (CSPs) containing CTF-specific response elements. Inhibition of Ca_V1 by specific calcium channel blockers (CCBs) antagonizes membrane depolarization and attenuates Ca^{2+}-dependent gene regulation. **(B, C) Selection of synthetic CSPs to quantify depolarization-dependent Ca_V1 signaling.** (B) $Ca_V1.2$ (p$Ca_V1.2$/ pKK56)- or (C) $Ca_V1.3$ (p$Ca_V1.3$:Δ42/pKK56)-transgenic HEK-293 cells were co-transfected with pWH31 (P_{cFOS}-

SEAP-pA), pHY30 (P_{NFAT1}-SEAP-pA), pMX57 (P_{NFAT3}-SEAP-pA) or pWH90 (P_{CRE2}-SEAP-pA) and cultivated in the presence or absence of 60 mM potassium chloride (KCl). SEAP levels in culture supernatants were scored after 48 h. Data are shown as mean ± SD (n=3 independent experiments).

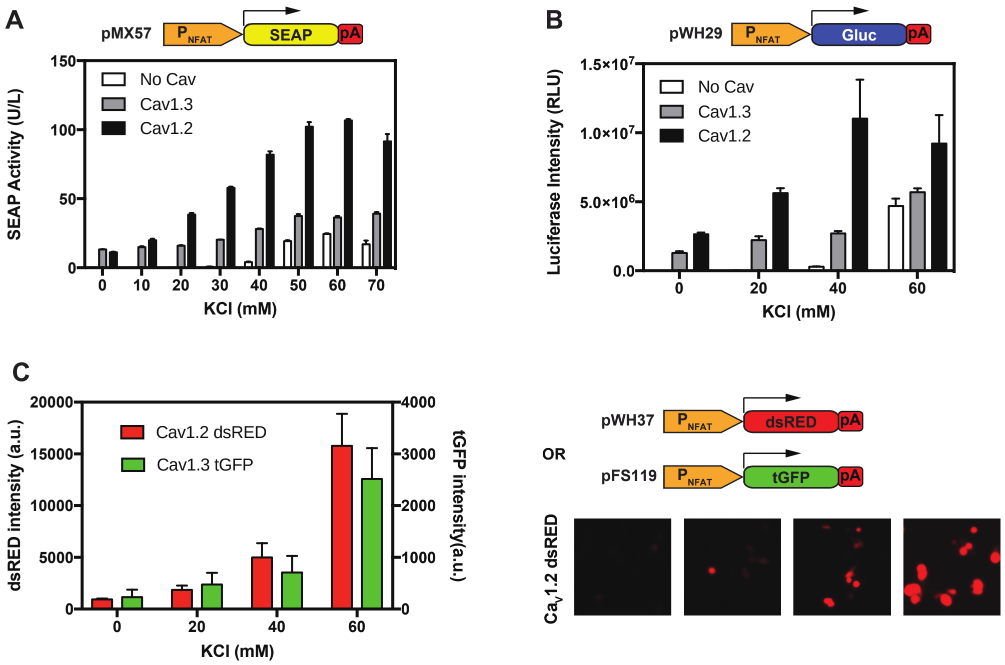

Figure S2. Depolarization-inducible reporter gene expression. (A) KCl-triggered SEAP expression. HEK-293 cells were co-transfected with pMX57 (P_{NFAT3}-SEAP-pA), pKK56 ($P_{hEF1\alpha}$-Cacna2d1-P2A-Cacnb3-pA) and either Ca_V1.2 (pCa_V1.2; P_{hCMV}-Cacna1c-pA), Ca_V1.3 (pCa_V1.3:Δ42; P_{hCMV}-Cacna1d:Δ42-pA) or no Ca_V (pcDNA3.1(+); P_{hCMV}-MCS-pA), and depolarized with KCl (0-70 mM). SEAP levels in culture supernatants were scored after 48 h. **(B) KCl-triggered GLuc expression.** Isogenic HEK-293 cells to (A) were transfected with a GLuc-expressing reporter vector (pWH29; P_{NFAT3}-Gluc-pA) instead of pMX57 and cultivated in cell culture medium containing different levels of KCl (0-60 mM). GLuc levels in culture supernatants were scored after 48 h. **(C) KCl-triggered expression of GFP and DsRed.** Isogenic HEK-293 cells to (A) were transfected with reporter vectors expressing DsRed (pWH37; P_{NFAT3}-DsRed-Express-pA) or tGFP (pFS119; P_{NFAT3}-TurboGFP:dest1-pA) instead of pMX57, and depolarized with KCl (0-60 mM). Fluorescence levels were recorded after 48 h by quantitative microplate reading (left; a.u., arbitrary unit) or microscopy (right). All data are presented as mean± SD (n=3 independent experiments).

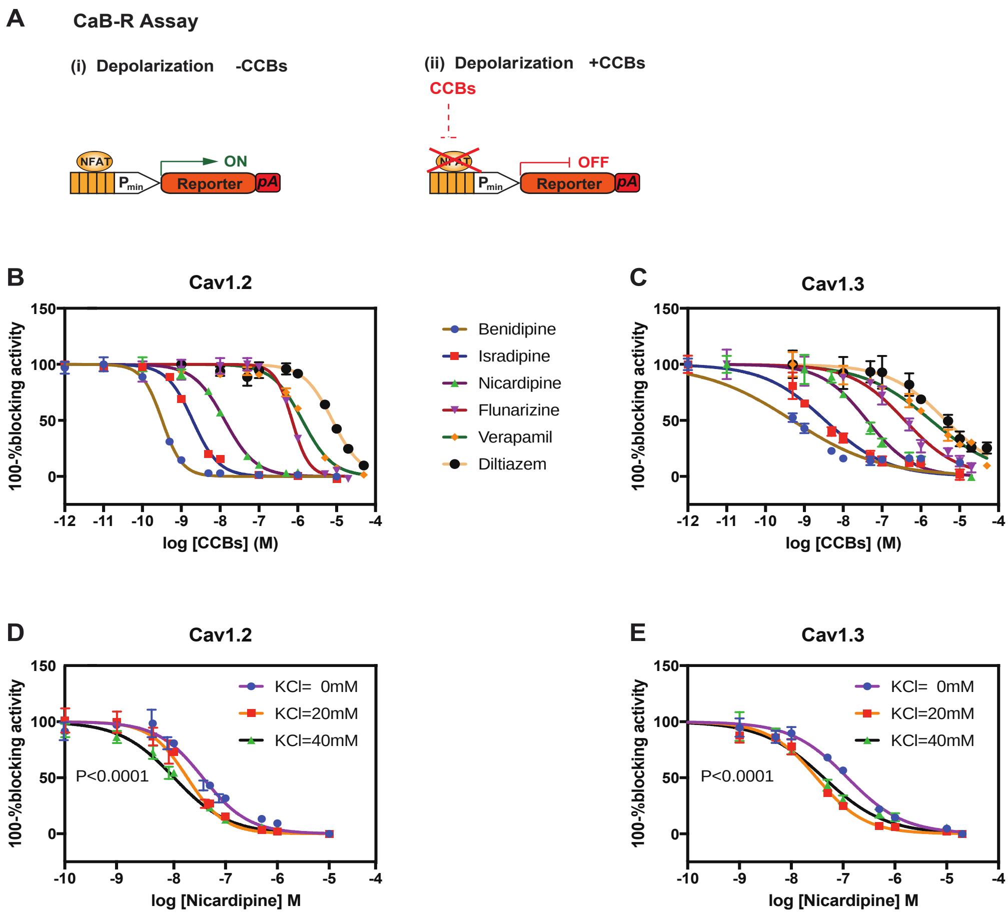

Figure S3. Design and validation of a calcium channel blocker-repressible (CaB-R) reporter assay. (A) Design principle. The presence of calcium channel blockers (CCBs) prevents reporter protein expression by inhibiting depolarization-dependent activation of endogenous NFAT (nuclear factor of activated T-cells) signaling. **(B, C) Validation of dose-dependent CaB-R with clinically approved CCBs.** (B) Ca$_V$1.2 (pCa$_V$1.2/pKK56/pMX57)- or (C) Ca$_V$1.3 (pCa$_V$1.3:Δ42/pKK56/pMX57)-transgenic HEK-293 cells were depolarized with 40 mM KCl and immediately placed in culture wells containing different concentrations of CCBs. **(D, E) Validation of CaB-R for analysis of use-dependent CCBs.** (D) Ca$_V$1.2 (pCa$_V$1.2/pKK56/pMX57)- or (E) Ca$_V$1.3 (pCa$_V$1.3:Δ42/pKK56/pMX57)-transgenic HEK-293 cells were depolarized with different levels of KCl (0, 20, 40 mM) and immediately added to culture wells containing different concentrations of nicardipine. SEAP levels in (B-E) were scored at 48 h after exposure to CCBs. Data are shown as mean percentage of relative blocking activity, normalized to maximal depolarization-dependent SEAP levels (0%; 40 mM KCl, no CCB) and maximal CCB blocking activity (100%; 10 µM nicardipine). Statistics of (D-E) were analyzed by means of an extra-sum-of-squares F test (n=3 independent experiments).

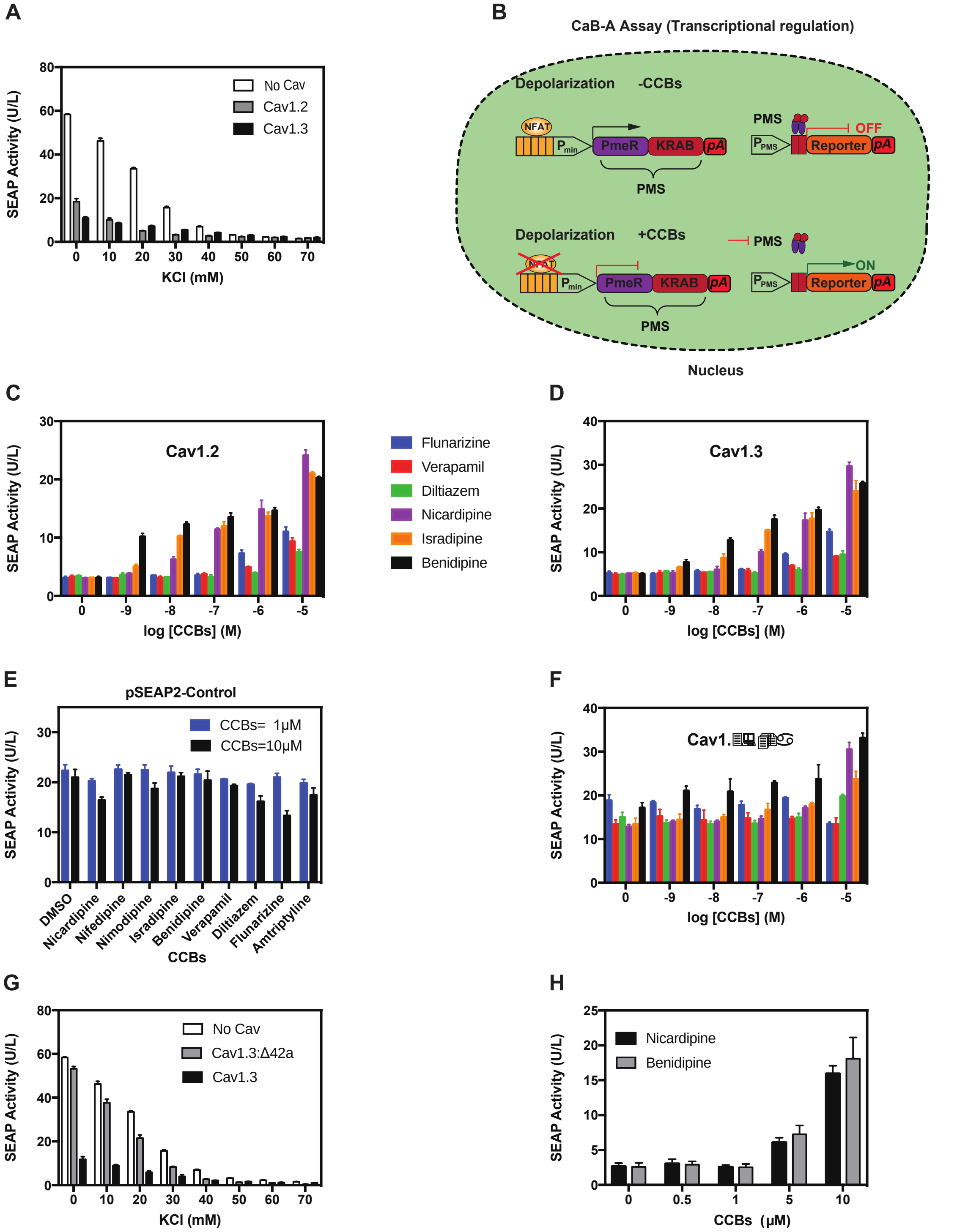

Figure S4. Design and validation of a calcium channel blocker-activated (CaB-A) reporter assay. (A) Depolarization-repressible reporter protein expression. HEK-293 cells were co-transfected with pWH5 (P_{PMS}-SEAP-pA), pWH75 (P_{NFAT4}-PMS-pA), pKK56 ($P_{hEF1\alpha}$-Cacna2d1-P2A-Cacnb3-pA) and either $Ca_V1.2$ (p$Ca_V1.2$; P_{hCMV}-Cacna1c-pA), $Ca_V1.3$ (p$Ca_V1.3$; P_{hCMV}-Cacna1d-pA) or no Ca_V (pcDNA3.1(+); P_{hCMV}-MCS-pA), and depolarized

with KCl (0-70 mM) and SEAP levels in culture supernatants were scored after 48 h. Data are presented as mean ± SD (n=3 independent experiments). **(B) Transcription-based CaB-A.** Synthetic NFAT-promoters control the production of the synthetic mammalian trans-silencer (PMS), which silences reporter gene transcription controlled by binding to synthetic cognate promoters (P_{PMS}). Calcium channel blockers (CCBs) activate reporter protein expression by inhibiting depolarization-dependent PMS-expression. **(C, D) Dose-dependent CaB-A activity.** (C) Ca_V1.2 (pCa_V1.2/pKK56/pWH75/pWH5)- or (D) Ca_V1.3 (pCa_V1.3/pKK56/pWH75/pWH5)-transgenic HEK-293 cells were depolarized with 30 mM KCl and immediately placed in culture wells containing different concentrations of CCBs. Data are shown as mean ± SD of SEAP levels scored at 48 h after exposure to CCBs (n=3 independent experiments). **(E) Cytotoxicity control of CCBs.** HEK-293 cells were transfected with pSEAP2-Control (P_{SV40}-SEAP-pA) and cultivated for 48 h in cell culture medium containing 1 µM or 10 µM CCBs. Data are shown as mean ± SD (n=3 independent experiments). **(F, G) Splice variant-dependent CaB-A activity.** (F) Ca_V1.3:Δ42-transgenic HEK-293 cells (pCa_V1.3:Δ42/pKK56/pWH75/pWH5) were depolarized with 30 mM KCl and immediately placed in culture wells containing different concentrations of CCBs. (G) HEK-293 cells expressing Ca_V1.3 (pCa_V1.3/pKK56/pWH75/pWH5), Ca_V1.3:Δ42 (pCa_V1.3:Δ42/pKK56/pWH75/pWH5) or no Ca_V (pcDNA3.1(+); P_{hCMV}-MCS-pA) were cultivated in cell culture medium containing different KCl levels (0-70 mM). Data in (F, G) are shown as mean ± SD of SEAP levels scored at 48 h after addition of KCl (n=3 independent experiments). **(H) Impact of CCBs on PMS activity.** HEK-293 cells were co-transfected with pWH9 (P_{SV40}-PMS-pA) and pWH5 (P_{PMS}-SEAP-pA), and cultivated in culture medium containing different concentration of nicardipine or benidipine. Data are shown as mean ± SD of SEAP levels scored at 48 h after exposure to CCBs (n=3 independent experiments).

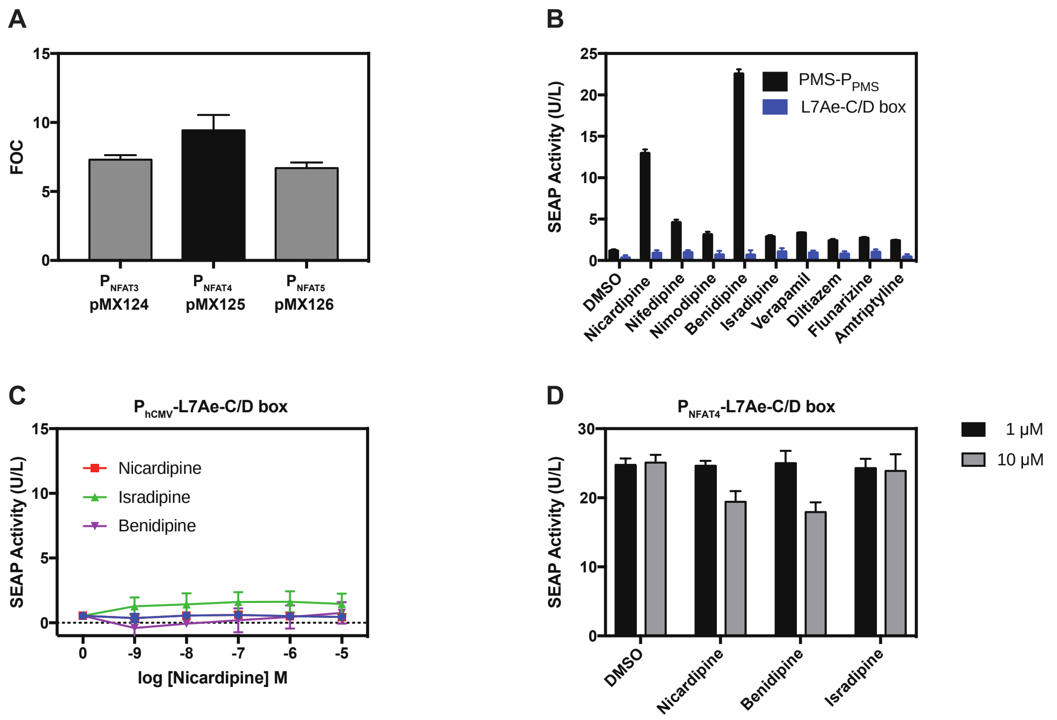

Figure S5. Control experiments of the translation-based (CaB-A) reporter assay. (A) Promoter optimization for NFAT-dependent L7Ae expression. HEK-293 cells were co-transfected with pCa$_V$1.2, pKK56, pMX199 and different L7Ae expression vectors containing five (pMX124; P$_{NFAT3}$-L7Ae-pA), seven (pMX125; P$_{NFAT4}$-L7Ae-pA) or nine (pMX126; P$_{NFAT5}$-L7Ae-pA) tandem repeats of NFAT response elements, depolarized with 20 mM KCl and immediately placed in culture wells containing 0 or 10 µM nicardipine. Data points are presented as mean FOC (fold of control, DMSO) of SEAP levels scored at 48 h after exposure to nicardipine (n=3 independent experiments). **(B-D) Impact of CCBs on NFAT-controlled repressors.** (B) Transcription- (PMS-P$_{PMS}$; pWH9/pWH5) or translation-based (L7Ae-C/D box; pWH145/pMX199; pWH145, P$_{hCMV}$-L7Ae-pA) control systems were cultivated for 48 h in cell culture medium containing 0 µM (DMSO control) or 10 µM CCBs. (C) pWH145/pMX199-transgenic HEK-293 cells were cultivated for 48 h in cell culture medium containing different concentrations of CCBs. (D) pMX125/pMX199-transgenic HEK-293 cells were cultivated for 48 h in cell culture medium containing 1 µM or 10 µM CCBs. Data in (B-D) are shown as mean ± SD of SEAP levels scored at 48 h after exposure to CCBs (n=3 independent experiments).

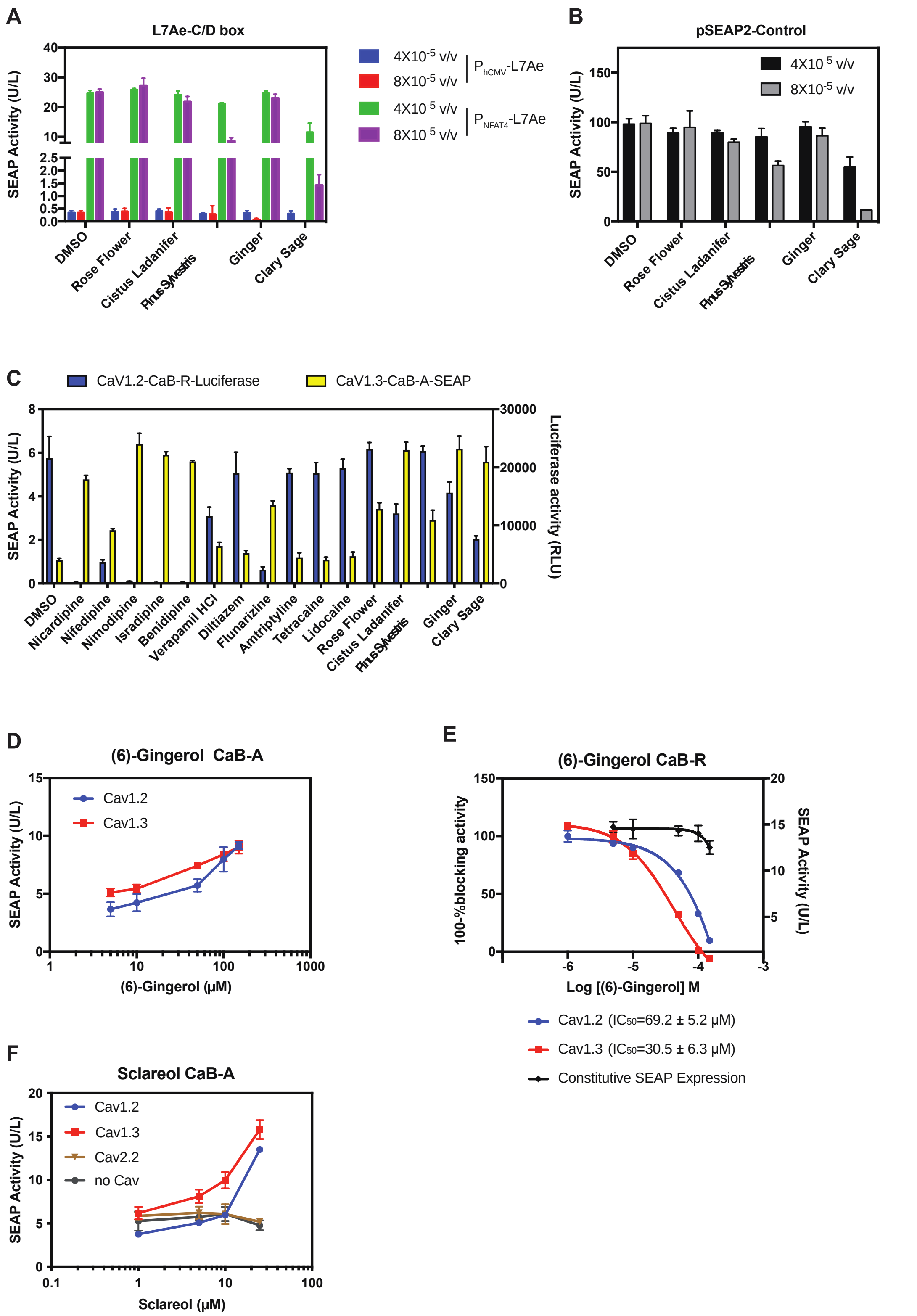

Figure S6. Identification of putative anti-Parkinson drug candidates. (A) Impact of plant essential oils on L7Ae-dependent control systems. pWH145/pMX199 (P_{hCMV}-L7Ae)- or pMX125/pMX199 (P_{NFAT4}-L7Ae)-transgenic HEK-293 cells were depolarized with 20 mM KCl and cultivated for 48 h in cell culture medium containing different levels of plant essential oils (4 x 10^{-5} or 8 x 10^{-5} v/v). **(B) Cytotoxicity control of plant essential oils.** HEK-

293 cells were transfected with pSEAP2-Control (P_{SV40}-SEAP-pA) and cultivated for 48 h in cell culture medium containing different levels of plant essential oils (4 x 10^{-5} or 8 x 10^{-5} v/v). All data are shown as mean ± SD of SEAP levels scored at 48 h after exposure to essential oils (n=3 independent experiments). **(C) Validation of CaB-A for multiplexed drug screening.** Independent Ca_V1.2-specific CaB-R (pCa_V1.2/pKK56/pWH29) and Ca_V1.3-specific CaB-A (pCa_V1.3/pKK56/pMX125/pMX199) systems were mixed, depolarized with 30 mM KCl, and added to culture wells supplemented with different plant essential oils (4x10^{-5} v/v) or CCBs (10 µM). Data are shown as mean ± SD of reporter proteins scored at 48 h after exposure to antagonists (n=3 independent experiments). Levels of the DMSO solvent (control) in cell culture medium were kept below 0.4%. **(D) Quantification of Ca_V1 antagonism by (6)-gingerol using CaB-A.** HEK-293 cells transfected with Ca_V1.2- or Ca_V1.3-dependent CaB-A were depolarized with 30 mM KCl and immediately seeded into culture wells containing different concentrations of (6)-gingerol. Data are shown as mean ± SD of SEAP levels scored at 48 h after drug exposure (n = 3 independent experiments). **(E) Quantification of Ca_V1 antagonism by (6)-gingerol using CaB-R.** HEK-293 cells transfected with Ca_V1.2- or Ca_V1.3-dependent CaB-R were depolarized with 30 mM KCl and immediately seeded into culture wells containing different concentrations of (6)-gingerol. HEK-293 cells transfected with a constitutive SEAP-expression vector (pSEAP2-Control; P_{SV40}-SEAP-pA) were used as a reference for putative cytotoxicity caused by drug exposure. Data are mean ± SD of SEAP levels scored at 48 h after drug exposure (n = 3 independent experiments). **(F) Quantification of Ca_V1 antagonism by sclareol using CaB-A.** HEK-293 cells transfected with Ca_V1.2- or Ca_V1.3-dependent CaB-A were depolarized with 30 mM KCl and immediately seeded into culture wells containing different concentrations of sclareol. HEK-293 cells transfected with a bacterial expression vector (pViM41; P_{T7}-mCherry-MCS) or Ca_V2.2 (pCa_V2.2; P_{hCMV}-Cacna1b-pA) instead of Ca_V1.2- or Ca_V1.3 were used as negative controls indicating Ca_V-unrelated or L-type Ca_V-unrelated assay readouts, respectively. Data are shown as mean ± SD of SEAP levels scored at 48 h after drug exposure (n = 3 independent experiments).

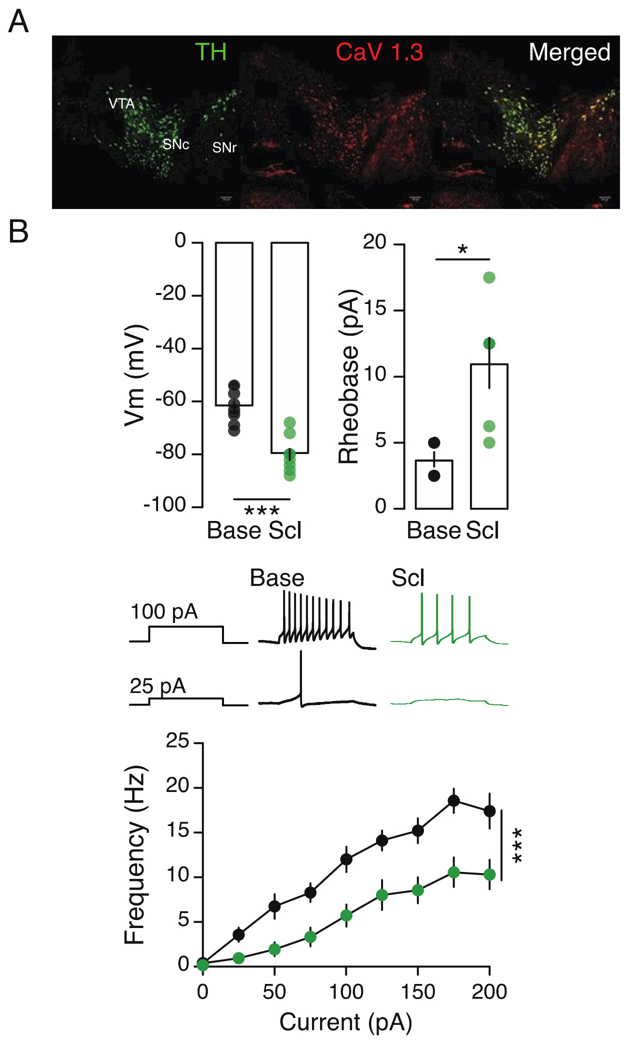

Figure S7. Sclareol hyperpolarizes SNc DA neurons. (A) Typical confocal image of midbrain-containing brain tissues stained for CaV1.3 (red) and TH (green) (n = 3 mice). **(B)** Sclareol bath application (10 µM) leads to SNc DA cell inhibition as revealed by neuronal hyperpolarisation (n = 9 cells) and a concomitant increase of rheobase (n = 7 cells) and downward shift of the firing activity (n=13 cells). Data are shown as the mean ± SEM, statistics by paired t-test (Vm and Rheobase) or two-way repeated-measures ANOVA test (input output curve). * $p<0.05$, *** $p<0.001$.

Movie S1. Concomitant locomotion tracking and striatal calcium dynamics of an example vehicle-treated mouse. The movies are sped up 4 times and show motor activity (upper videos) and striatal calcium activity (lower videos). These were recorded on the baseline session (left) and day 30 of the experiment (right).

Movie S2. Concomitant locomotion tracking and striatal calcium dynamics of an example sclareol-treated mouse. The movies are sped up 4 times and show motor (upper videos) and striatal calcium activities (lower videos). These were recorded on the baseline session (left) and day 30 of the experiment (right).

Supplementary Tables

Table S1. IC_{50} values of FDA-approved calcium channel blockers (CCBs) on $Ca_V1.2$ and $Ca_V1.3$

**Class**	**Compound**	**IC_{50} ($Ca_V1.2$)**			**IC_{50} ($Ca_V1.3$)**		
		Literature range	Physiological KCl=0 mM (nM)	Depolarized KCl=40 mM (nM)	Literature range	Physiological KCl=0 mM (nM)	Depolarized KCl=40 mM (nM)
Dihydropyridine	Nicardipine	13-160nM[1-4]	45.8±9.0	12.7±2.3	n/A	112±28.0	41.4±13.5
	Nimodipine	3-139nM[4,5]	33.2±7.1	15.7±1.9	2.7-3μM[5,6]	16.5±3.6	3.0±1.6
	Isradipine	0.4-29.7nM[5,7]	4.7±0.6	1.9±0.2	0.5-30nM[5,7]	18.4±4.6	3.5±1.4
	Benidipine	n/A	0.73±0.1	0.28±0.03	n/A	3.7±1.1	0.49±0.45
Phenylalkylamine	Verapamil	0.7 – 35.3μM[1,4]	6527±801	1263±208	199μM[8]	6179±1315	1836±652
Benzothiazepine	Diltiazem	1.8-33.0μM[1,2,4,5]	9859±1359	6238±1591	326μM[8]	13340±2570	4387±2159
Nonselective	Flunarizine	0.1–11μM[9]	1086±235	531.3±47.4	0.1–11μM[9]	562±187	387±163
Others	Amitriptyline	23.2μM[10]	8872±1087	4688±933	23.2μM[10]	7815±1797	9169±2224

Values are mean ±SD of 3 independent experiments.

Table S2. Essential oils used in this manuscript

**Essential oil**	**Source**
1 Caraway	Golgemma, Esperaza, France
2 Nutmeg	Golgemma, Esperaza, France
3 Peppermint	Primavera Life GmbH, Oy-Mittelberg, Germany
4 Rosemary	Vie Arôme, Montreuil, France
5 Petit Grain	Golgemma, Esperaza, France
6 Lemon	Golgemma, Esperaza, France
7 Savory	Golgemma, Esperaza, France
8 Neroli	Golgemma, Esperaza, France
9 Rose Flower	Vie Arôme, Montreuil, France
10 Cajeput	Golgemma, Esperaza, France
11 Coriander	Vie Arôme, Montreuil, France
12 Geranium Bourbon	Welfine Beijing Science & Technology Ltd, Beijing, China
13 Cistus Ladanifer	Welfine Beijing Science & Technology Ltd, Beijing, China
14 Thyme Thymol	Welfine Beijing Science & Technology Ltd, Beijing, China
15 Marjoram	Vie Arôme, Montreuil, France
16 Cinnamon Bark	Vie Arôme, Montreuil, France
17 Pinus Sylvestris	Vie Arôme, Montreuil, France
18 Bitter Orange	Vie Arôme, Montreuil, France
19 Cumin	Golgemma, Esperaza, France
20 Rosewood	Vie Arôme, Montreuil, France
21 Sandalwood	Golgemma, Esperaza, France
22 Ginger	Primavera Life GmbH, Oy-Mittelberg, Germany
23 Maritime Pine	Golgemma, Esperaza, France
24 Camomile	Golgemma, Esperaza, France
25 Basil	Vie Arôme, Montreuil, France
26 Clove	Primavera Life GmbH, Oy-Mittelberg, Germany
27 Verbena	Golgemma, Esperaza, France
28 Clary Sage	Vie Arôme, Montreuil, France
29 Cupressus	Vie Arôme, Montreuil, France
30 Spearmint	Primavera Life GmbH, Oy-Mittelberg, Germany

31 Palma Rosa	Golgemma, Esperaza, France
32 Oregano	Golgemma, Esperaza, France
33 Juniper	Vie Arôme, Montreuil, France
34 Lemongrass	Golgemma, Esperaza, France
35 Thyme Vulgaris	Welfine Beijing Science & Technology Ltd, Beijing, China
36 Winter Savory	Welfine Beijing Science & Technology Ltd, Beijing, China
37 Bay tree	Golgemma, Esperaza, France
38 Clove nutmeg	Golgemma, Esperaza, France
39 Fine Lavender	Golgemma, Esperaza, France
40 Myrtle	Golgemma, Esperaza, France
41 Ylang-ylang	Vie Arôme, Montreuil, France
42 Thyme Linalool	Vie Arôme, Montreuil, France

Table S3. Reference compounds for the generation of $Ca_V1.3$-inhibiting pharmacophores

**Positive reference (known Ca_V1 blockers)**	**Negative reference (Ca_V-independent blockers)**
Benidipine	Amtriptyline
Cilnidipine	Lidocaine
Nimordipine	Tetracaine
Isradipine	
Nicardipine	
Nifedipine	
Flunarizine	
Verapamil	
Diltiazem	
Pyrimidine-2,4,6,-trione	
1-(3-chlorophenethyl)-3-cyclopentylpyrimidine-2,4,6-(1H,3H,5H)-trione	

Table S4. Virtual Screening Experiment

a. 309 compounds from 5 essential oils:

SMILES	Chemical Name
**Ciste essential oil**	
CC1=CCC2CC1C2(C)C	A-PINENE
CC1(C2CC1C(=C)C(C2)O)C	(-)-TRANS-PINOCARVEOL
CC1CCC2C1C3C(C3(C)C)CCC2(C)O	LEDOL
CC(=O)C1=CC=CC=C1	ACETOPHENONE
CC(=O)OC1CC2CCC1(C2(C)C)C	BORNYL ACETATE
CC1=CCC(CC1)(C(C)C)O	TERPINEN-4-OL
CC1=CC=C(C=C1)C(C)C	P-CYMENE
CC1CC(=NO)CC(C1)(C)C	3,3,5-Trimethylcyclohexanone
CC(=O)OC1(CCCC(C1=O)(C)C)C	2-Acetoxy-2,6,6-trimethylcyclohexanone
CC1(C2CCC(C2)(C1O)C)C	1,3,3-Trimethylbicyclo[2.2.1]heptan-2-ol
CC1(C2CCC(C2)(C1=O)C)C	1,3,3-Trimethylbicyclo[2.2.1]heptan-2-one
CC1(C2CCC1(C(=O)C2)C)C	Camphor 1,7,7-trimethylbicyclo[2.2.1]heptan-2-one
CC1(C2CCC1(C(C2)O)C)C	BORNEOL
CC1=CC(=O)C2CC1C2(C)C	(-)-VERBENONE
CC1(C2CC=C(C1C2)CO)C	MYRTENOL
CC1=CCC(CC1)C(=C)C	LIMONENE
CC1(CCC(O1)C(C)(C)O)C=C	LINALOOL OXIDE
CC1=CC=C(C=C1)C(C)(C)O	8-HYDROXY-P-CYMENE
CC1=CC=C(C=C1)C(C)C	DIHYDRO P-CYMENE
C1=CC=C(C=C1)CCO	CC1=CC=C(C=C1)C(C)C
CC1(C2CC=C(C1C2)C=O)C	MYRTENAL
CC1CCC2C1C3C(C3(C)C)CCC2=C	ALLOAROMADENDRENE
CC1(C2CCC(C2)C1=C)C	CAMPHENE
CC(C)C1CCC2(C3C1C4C2(C4C3)C)C	(+)-CYCLOSATIVENE
CC1=CC2C(CCC(=C2CC1)C)C(C)C	(+)-DELTA-CADINENE
CC1=CCC(C=CCC(=CCC1)C)(C)C	humulene
CC1CCC23C1C4C(C4(C)C)CCC2(O3)C	ledene oxide
CCCCCC=CC(=O)C	trans-3-Nonen-2-one
CC(=CCCC(C)(C=C)O)C	LINALOOL
CC1(C2CCC(O1)(CC2)C)C	EUCALYPTOL
CC1=CCC(CC1=O)C(=C)C	CARVONE
CC1CCC(CC2=C1CCC2C)C(=C)C	A-GUAIENE

CC(CCCC(C)(C)O)C=C	DIHYDROMYRCENOL
CC(=O)OCC1=CCC2CC1C2(C)C	MYRTENYL ACETATE
CC1=CCC2C3C1C2(CCC3C(C)C)C	COPAENE
CC1=CC(=O)CC(C1)(C)C	ISOPHORONE
CC1=C(C=CC(=C1)C(=O)C)O	4-Hydroxy-3-methylacetophenone
CC1=CC=C(C=C1)C(=O)C	P-METHYLACETOPHENONE
COC1=C(C=CC(=C1)CC=C)O	EUGENOL
CC(CCC=C(C)C)CCO	CITRONELLOL
CC1=CC(=C(C=C1)C(C)C)O	THYMOL
CC(C)C1=CC=C(C=C1)CO	P-CYMEN-7-OL
CC1CCOC(C1)C=C(C)C	ROSE OXIDE
CC1(C2CCC(=C)C1C2)C	B-PINENE
CC(=CCCC(=O)C)C	6-Methyl-5-hepten-2-one
CC1(C2CC3C1(C3C2)C)C	TRICYCLENE
CC1=CCC(=C(C)C)CC1	Terpinolene
CC1=CCC(=CC1)C(C)C	GAMMA-TERPINENE
CC1(C2CCC(O1)(CC2)C)C	EUCALYPTOL
CC1=CCC(C=C1)C(C)C	ALPHA-PHELLANDRENE
**Ginger essential oil**	
CCCCCC(CC(=O)CCC1=CC(=C(C=C1)O)OC)O	6-Gingerol
CCCC(CC(=O)CCC1=CC(=C(C=C1)O)OC)O	(4)-Gingerol
CCCCCCCCCC(CC(=O)CCC1=CC(=C(C=C1)O)OC)O	(10)-Gingerol
CCCCCCCC(CC(=O)CCC1=CC(=C(C=C1)O)OC)O	(8)-Gingerol
CCCCCC=CC(=O)CCC1=CC(=C(C=C1)O)OC	6-Shogaol
CC1=CCC(C=C1)C(C)CCC=C(C)C	l-Zingiberene
CC(CCC=C(C)C)C1CCC(=C)C=C1	B-sesquiphellandrene
CC1=CCC(CC1)C(=C)CCC=C(C)C	B-BISABOLENE
CC1(C2CCC(C2)C1=C)C	(+)-Camphene
CC1(C2CCC(C2)C1=C)C	(-)-Camphene
CC1=CC=C(C=C1)C(C)CCC=C(C)C	A-CURCUMENE
CC(C)C1CCC(=C)C=C1	B-PHELLANDRENE
CC(=CCCC(=CCC=C(C)C=C)C)C	A-FARNESENE
CC1=CC2C(CC1)C(=C)CCC2C(C)C	GAMA-MUUROLENE
CC1(C2CCC(O1)(CC2)C)C	EUCALYPTOL
CC1=CCC2CC1C2(C)C	A-PINENE
CC1CCC(C2C13C2C(=CC3)C)C(C)C	Alpha-Cubebene
CC1CCC(C2C13C2C(=C)CC3)C(C)C	Beta-Cubebene
CC1=CCC(CC1)C(=C)C	(+)LIMONENE
CC1(C2CCC1(C(C2)O)C)C	ENDO-BORNEOL
CC(=C)[C@@H]1CC[C@@]([C@@H](C1)C(=C)C)(C)C	B-ELEMENE

=C	
CCCC=C(C)C(=O)C	3-Methylhept-3-en-2-one
CC(=CCCC(=O)C)C	Methyl Heptenone
CC(=CCCC(=C)C=C)C	MYRCENE
CC(=C1CCC(C(C1)C(=C)C)(C)C=C)C	GAMA-ELEMENE
CC1=CCC(CC1)C(C)(C)O	A-TERPINEOL
CCCCCC=O	HEXANAL
CC1=CCC2C3C1C2(CCC3C(C)C)C	COPAENE
CC(=C)C1CC(CCC1(C)C=C)C(C)(C)O	ELEMOL
CC(=CCCC(=CCCC(C)(C=C)O)C)C	NEROLIDOL
CC(=CCCC(=CCOC(=O)C)C)C	GERANYL ACETATE
CC1=CCCC2(C1CC(CC2)C(=C)C)C	A-SELINENE
CC1CCC2C1C3C(C3(C)C)CCC2=C	ALLOAROMADENDRENE
CC(C)C1CCC2(C3C1C4C2(C4C3)C)C	(+)-CYCLOSATIVENE
CC1=CCC(CC1)(C(C)CCC=C(C)C)O	B-BISABOLOL
CCCCCCCCCC=O	DECANAL
CC1(C2CCC(=C)C1C2)C	B-PINENE
CC12CCCC(=C)C1CC(CC2)C(C)(C)O	B-EUDESMOL
CC(=CCC/C(=C/CO)/C)C	GERANIOL
CC1=CCC(C=C1)C(C)C	A-PHELLANDRENE
CC(=CCCC(C)(C=C)O)C	LINALOOL
CC(=C1CCC(C(C1)C(=C)C)(C)C=C)C	GAMA-ELEMENE
CCCCCCCC=O	OCTANAL
CCCCCCCCCC(=O)C	METHYL NONYL KETONE
CC1=CCC(=C(C)C)CC1	TERPINOLENE
CC1=CC(=C(C(=C1C)O)C)C	2,3,5,6-TETRAMETHYL PHENOL
CC(=CCCC1(C2CCC(=C)C1C2)C)C	BERGAMOTENE
CC1(C2CC3C1(C3C2)C)C	TRICYCLENE
CC1=CCCC2(C1CC(CC2)C(C)(C)O)C	A-EUDESMOL
CC(=O)OC1CC2CCC1(C2(C)C)C	BORNYL ACETATE
CCCCCC(=O)C	2-HEPTANONE
CC(C)C1CCC(C(C1O)C(=C)C)(C)C=C	6-SHYOBUNOL
CC1(C2CCC1(C(=O)C2)C)C	CAMPHOR
CC(=CCCC(=CC=O)C)C	B-CITRAL
CC1=CC2C(CCC(C2CC1)(C)O)C(C)C	A-CADINOL
CC1=CC=C(C=C1)C(C)C	P-CYMENE
CC(C)C12CCC(=C)C1C2	SABINENE
CC1CCC(C2=C1C=CC(=C2)C)C(C)C	CALAMENENE
CC1CCC(C2C13C2C(=CC3)C)C(C)C	CUBEBENE
CC1=CC=CC=C1	TOLUENE
CC1=CCC2C3C1C2(CCC3C(C)C)C	B-YLANGENE
CC(CCC=C(C)C)CC=O	CITRONELLAL

CCCCCCCCCCCC(=O)C	2-TRIDECANONE
CCCCCCCC(=O)C	2-NONANONE
CC1CCC=C2C1(C3C(C3(C)C)CC2)C	(+)-CALARENE
CC1=C2CC(CCC2(CCC1)C)C(C)(C)O	GAMA-EUDESMOL
CC(=CCCC(=CCCC(=O)C)C)C	GERANYL ACETONE
CC1CCC(C2C13C2C(=CC3)C)C(C)C	A-CUBEBENE
CCCCCC(C)O	2-HEPTANOL
CC1(C2CC=C(C1C2)C=O)C	(-)-MYRTENAL
CC1=CCC(=CC1)C(C)C	GAMA-TERPINENE
CC1=CCC2C(C1)C2(C)C	3-CARENE
CC(=C)C1CCC(=C)CC1	p-Mentha-1(7),8-diene
CC(=CCCC1=COC=C1)C	PERILLEN
CCCCCCCC(C)O	2-NONANOL
CC1(CC2C1CCC3(C(O3)CCC2=C)C)C	CARYOPHYLLENE OXIDE
CC1=CCC(C2=C1C=CC(=C2)C)C(C)C	A-CALACORENE
CC1=CC=C(CC1)C(=C)C	1,3,8-p-Menthatriene
CC(=O)CCC1=CC(=C(C=C1)O)OC	VANILLYLACETONE
CCCCC=C(C)C	2-METHYL-2-HEPTENE
CC1=CCC(CC1)C(=C)CCC=C(C)C	B-OCIMENE
CCCCC	PENTANE
**Pine essential oil**	
CC1(C2CCC(=C)C1C2)C	B-PINENE
CC1=CCC2CC1C2(C)C	A-PINENE
CC1=CCC(CC1)C(=C)C	LIMONENE
CC1=CC2C(CCC(=C2CC1)C)C(C)C	(+)-DELTA-CADINENE
CC1(C2CCC(C2)C1=C)C	CAMPHENE
CC1=CCC(CC1)C(C)(C)O	A-TERPINEOL
CC1=CCCC(=C)C2CC(C2CC1)(C)C	B-CARYOPHYLLENE
CC(=CCCC(=C)C=C)C	MYRCENE
CC1CCC(C2C13C2C(=CC3)C)C(C)C	alpha-Cubebene
CC(C)C1CCC(=C)C=C1	B-PHELLANDRENE
CC1=CC2C(CC1)C(=CCC2C(C)C)C	A-MUUROLENE
CC1=CCC(C=CCC(=CCC1)C)(C)C	HUMULENE
CC1=CCC(=C(C)C)CC1	TERPINOLENE
CC1=CCC2C(C1)C2(C)C	3-CARENE
CC1=CCC(CC1)C(=C)CCC=C(C)C	B-OCIMENE
CC1CCC(C2C13C2C(=C)CC3)C(C)C	beta-Cubebene
CC1(C2CC3C1(C3C2)C)C	TRICYCLENE
CC1(C2CC1C(=C)C(C2)O)C	TRANS-PINOCARVEOL
CC(=O)OC1CC2CCC1(C2(C)C)C	BORNYL ACETATE
CC(=O)OC1C(C2CCC1(C2)C)(C)C	Fenchyl Acetate
CC1CCC2C13CCC(C(C3)C2(C)C)(C)O	CEDROL
CC1(C2CCC1(C(=O)C2)C)C	CAMPHOR

CC1(C2CCC1(C(C2)O)C)C	BORNEOL
CC1(C2CC=C(C1C2)CO)C	MYRTENOL
CC1(C2CC=C(C1C2)C=O)C	MYRTENAL
CC1(CCCC2(C3C1C(C2=C)CC3)C)C	LONGIFOLENE
CC(=C1CCC(C(C1)C(=C)C)(C)C=C)C	GAMA-ELEMENE
CC1=CC2C(CCC(C2CC1)(C)O)C(C)C	A-CADINOL
CC1C2CC(C2(C)C)CC1=O	3-PINANONE
CC1=CC=C(C=C1)C(C)C	P-CYMENE
CC1(CC2C1CCC3(C(O3)CCC2=C)C)C	CARYOPHYLLENE OXIDE
CC(C)C1CCC2(C3C1C2C(=C)CC3)C	B-YLANGENE
CC1=CCC2C3C1C2(CCC3C(C)C)C	COPAENE
CC=CC1=CC(=C(C=C1)OC)OC	METHYL ISOEUGENOL
CC(C)C12CCC(=C)C1C2	SABINENE
CC1=CCC(=CC1)C(C)C	GAMA-TERPINENE
CC1CCC(C2C13C2C(=CC3)C)C(C)C	A-CUBEBENE
CC1(C2CCC(C2)(C1=O)C)C	FENCHONE
CCCCCC=O	HEXANAL
CC1CC2C(C2(C)C)C=C1	4-CARENE
CC1=CCC(C=C1)C(=C)C	DIHYDRO P-CYMENE
CC1=CCC(C1(C)C)CC=O	A-CAMPHOLENAL
CC(C)C1CCC2(C1C3C2CCC3=C)C	B-BOURBONENE
C1=CC=C(C=C1)CCO	PHENETHYL ALCOHOL
CCCCCC1=CC=CO1	2-PENTYLFURAN
CC1=CCC(C=C1)C(C)C	A-PHELLANDRENE
CC1=CCC2C3C1C2(CCC3C(C)C)C	A-YLANGENE
CC(=O)CCC1=CC=CC=C1	4-PHENYL-2-BUTANONE
C1=CC=C(C=C1)C=O	BENZALDEHYDE
CCCCCCCCC=O	NONANAL
CCOC(=O)C1=CC=CC=C1C(=O)OCC	DIETHYL PHTHALATE
**Rose Damascus essential oil**	
CC(CCC=C(C)C)CCO	CITRONELLOL
CC(=CCCC(=CCO)C)C	GERANIOL
CCCCCCCCCCCCCCCCCCC	NONADECANE
CC(=CCCC(=CCO)C)C	NEROL
CCCCCCCCCCCCCCCCCCCCC	HENEICOSANE
CCCCCCCCCC=CCCCCCCCC	9-NONADECENE
CCCCCCCCCCCCCCCCC	HEPTADECANE
CC(=CCCC(=CCCC(=CCO)C)C)C	FARNESOL
COC1=C(C=CC(=C1)CC=C)O	EUGENOL
CC(=CCCC(C)(C=C)O)C	LINALOOL
C1=CC=C(C=C1)CCO	PHENETHYL ALCOHOL
CCCCCCCCCCCCCCCCCCCCCCCCCC	HEXACOSANE
CC(=CCCC(=CCOC(=O)C)C)C	GERANYL ACETATE

COC1=C(C=C(C=C1)CC=C)OC	METHYLEUGENOL
CCCCCCCCCCCCCCCCCCCC	EICOSANE
CC(=CCCC(=CC=O)C)C	CITRAL
CC1=CCC2CC1C2(C)C	A-PINENE
CC(=CCCC(=CC=O)C)C	B-CITRAL
CC(CCC=C(C)C)CCOC(=O)C	CITRONELLYL ACETATE
CC1=CCC(CC1)C(C)(C)O	A-TERPINEOL
CC1CCOC(C1)C=C(C)C	ROSE OXIDE
CC1CCC(CC2=C1CCC2C)C(=C)C	A-GUAIENE
CC(=CCCC(=C)C=C)C	MYRCENE
CC1=CCC(C=CCC(=CCC1)C)(C)C	HUMULENE
CC1CCC2=C(CCC(CC12)C(=C)C)C	A-BULNESENE
CCCCCCCCCCCCCCCCCC	OCTADECANE
CCCCCCCCCCCCCCC	PENTADECANE
CC(=O)OCCC1=CC=CC=C1	PHENETHYL ACETATE
CCCCCCCCCCCCCCCCCCCCCCCCCCCC	OCTACOSANE
CCCCCCCCC=CCCCCCCC	8-HEPTADECENE
CCCCCCCCCCC=CCCCCCCCCC	10-HENEICOSENE
CC1(C2CCC(=C)C1C2)C	B-PINENE
CCCCCCCCCCC=CCCCCCCCC	9-EICOSENE
CC(=CCCC(=CCCC(C)(C=C)O)C)C	NEROLIDOL
CCCCCCC=O	HEPTANAL
CCCCCCO	HEXANOL
CC(=CCCC(=CCC=C(C)C=C)C)C	A-FARNESENE
CCCCCCCCCCCCCCCC	HEXADECANE
CC12CCCC(=C)C1CC(CC2)C(C)(C)O	B-EUDESMOL
CC(=CCCC(=CCCC(=CC=O)C)C)C	FARNESAL
CC1=CCCC2(C1CC(CC2)C(C)(C)O)C	A-EUDESMOL
CC(C)C1CCC2(C1C3C2CCC3=C)C	B-BOURBONENE
C1=CC=C(C=C1)C=O	BENZALDEHYDE
C1=CC=C(C=C1)COC(=O)C2=CC=CC=C2	BENZYL BENZOATE
CC1=CCC(=CC1)C(C)C	GAMA-TERPINENE
C1=CC=C(C=C1)CCOC(=O)C2=CC=CC=C2	PHENETHYL BENZOATE
CC1=CCOC(C1)C=C(C)C	NEROL OXIDE
CCCCCCCCC=O	NONANAL
CC1=CCC(CC1)C(=C)C	D-LIMONENE
CC1=CCC(CC1)C(=C)C	L-LIMONENE
CC(=CCC=C(C)C=C)C	B-OCIMENE
CCC(C)CO	2-METHYL-1-BUTANOL
CCCCCCCCCCCCCCCCCC=C	1-NONADECENE
CCCCC=O	PENTANAL
CCC(C)C(=O)OCCC1=CC=CC=C1	2-PHENYLETHYL 2-METHYLBUTYRATE

CC1=CCC(=C(C)C)CC1	TERPINOLENE
CC1(CC2C1CCC3(C(O3)CCC2=C)C)C	CARYOPHYLLENE OXIDE
CC(C)C12CCC(=C)C1C2	SABINENE
CC1CC2C(C2(C)C)C=C1	4-CARENE
CC(CCC=C(C)C)CCOC=O	CITRONELLYL FORMATE
CC1(C2CCC(O1)(CC2)C)C	EUCALYPTOL
CCC=CCCO	LEAF ALCOHOL
CC1=CC=C(C=C1)C(C)C	P-CYMENE
CC(=CCCC(=O)C)C	6-METHYL-5-HEPTEN-2-ONE
CC(=CCC=C(C)C=C)C	TRANS-B-OCIMENE
CC1(CCC(O1)C(C)(C)O)C=C	LINALOOL OXIDE
CCCCCO	1-PENTANOL
CCCCCC=O	HEXANAL
CC(C)CCCC(C)CCO	3,7-DIMETHYL-1-OCTANOL
C1=CC=C(C=C1)CO	BENZYL ALCOHOL
CCCCCCCO	HEPTANOL
CC(CCC=C(C)C)CC=O	CITRONELLAL
CC1=CCC(C=C1)C(C)C	A-PHELLANDRENE
CCCCCCCC=O	OCTANAL
CCCCCCCCCCCCC	TRIDECANE
CCCCCCCCO	OCTANOL
CC1(C2CCC(C2)C1=C)C	CAMPHENE
CCCCCCCC	OCTANE
CCC(C)O	2-BUTANOL
CCCCCCCCCC	DECANE
CCCC=O	BUTANAL
**Clary Sage essential oil**	
CC(=CCCC(C)(C=C)OC(=O)C)C	LINALYL ACETATE
CC(=CCCC(C)(C=C)O)C	LINALOOL
CC1=CCC2(CCCC(C23C1C3)(C)C)C	CIS-THUJOPSENE
CC1=CCCC(=C)C2CC(C2CC1)(C)C	B-CARYOPHYLLENE
CC1CCC2C13CC=C(C(C3)C2(C)C)C	(-)-ALPHA-CEDRENE
CC1=CCC(CC1)C(C)(C)O	A-TERPINEOL
CC(=CCCC(=CCOC(=O)C)C)C	GERANYL ACETATE
CC1CCC(C2C13C2C(=CC3)C)C(C)C	Alpha-Cubebene
CC1CCC(C2C13C2C(=C)CC3)C(C)C	Beta-Cubebene
CC1CCC2C13CCC(C(C3)C2(C)C)(C)O	CEDROL
CC(=CCCC(=CCOC(=O)C)C)C	NERYL ACETATE
CC(=CCC/C(=C/CO)/C)C	GERANIOL
CC1=CCC2C3C1C2(CCC3C(C)C)C	A-COPAENE
CC1=CCC(CC1)C(=C)C	LIMONENE

CC1(CC2C1CCC3(C(O3)CCC2=C)C)C	CARYOPHYLLENE OXIDE
CC1(CCCC2(C1CCC(C2CCC(C)(C=C)O)(C)O)C)C	SCLAREOL
CC(=C)CCCC(=C)C=C	alpha.-Myrcene
CC(=CCCC(=C)C=C)C	Beta-Myrcene
CC(=CCCC(=CCO)C)C	NEROL
CC1(C2CCC1(C(=O)C2)C)C	CAMPHOR
CC1=CCC2CC1C2(C)C	A-PINENE
CC(=CCC=C(C)C=C)C	B-OCIMENE
CC1=CCC(C=CCC(=CCC1)C)(C)C	HUMULENE
CC1(C2CCC(O1)(CC2)C)C	EUCALYPTOL
CC(C)C1CCC2(C1C3C2CCC3=C)C	(-)B-BOURBONENE
CC1(C2C1C3C(CCC3(C)O)C(=C)CC2)C	SPATHULENOL
CCCCCCC(C)C	2-METHYLOCTANE
CC1=CC=C(C=C1)C2(CCCC2(C)C)C	(+)CUPARENE
CC1(C2CCC1(C(C2)O)C)C	ENDO-BORNEOL
CC1(C2CCC(=C)C1C2)C	B-PINENE
CC(=CCC=C(C)C=C)C	TRANS-B-OCIMENE
CC1(CCCC2(C1=CCC(CC2)(C)O)C)C	WIDDROL
CC1(CCC(O1)C(C)(C)O)C=C	LINALOOL OXIDE
CC1=CCC2C3(CCCC(C3CCC2(O1)C)(C)C)C	SCLAREOLOXIDE
CC1(CC2C1CCC3(C(O3)CCC2=C)C)C	CARYOPHYLLENE OXIDE
CC1(CCC(O1)C(C)(C)O)C=C	LINALOOL OXIDE
CC(=CCCC(=CCC=C(C)C=C)C)C	A-FARNESENE
CCC=CCCO	LEAF ALCOHOL
CCCCCCC=O	HEPTANAL
CC1=CCC(=C(C)C)CC1	TERPINOLENE
CCCCCCOC=O	HEXYL FORMATE
CCCC=CCO	TRANS-2-HEXENOL
CC1=CC=C(C=C1)C(C)C	P-CYMENE
CC(C)C1CCC(=C)C=C1	B-PHELLANDRENE
CC1(C2CCC(C2)C1=C)C	COMPHENE

b. LigandScout result

**Screening Results:**	
CC(=O)CCC1=CC(=C(C=C1)O)OC	VANILLYLACETONE
CCCC(CC(=O)CCC1=CC(=C(C=C1)O)OC)O	(4)-Gingerol
CCCCCC=CC(=O)CCC1=CC(=C(C=C1)O)OC	6-Shogaol
CCCCCC(CC(=O)CCC1=CC(=C(C=C1)O)OC)O	6-Gingerol
CCCCCCCC(CC(=O)CCC1=CC(=C(C=C1)O)OC)O	(8)-Gingerol
CCOC(=O)C1=CC=CC=C1C(=O)OCC	DIETHYL PHTHALATE
CCCCCCCCCC(CC(=O)CCC1=CC(=C(C=C1)O)OC)O	(10)-Gingerol
CC1(CCCC2(C1CCC(C2CCC(C)(C=C)O)(C)O)C)C	SCLAREOL

CC1=C(C=CC(=C1)C(=O)C)O	4-Hydroxy-3-methylacetophenone
CC1(CCC(O1)C(C)(C)O)C=C	LINALOOL OXIDE
CC=CC1=CC(=C(C=C1)OC)OC	METHYL ISOEUGENOL
COC1=C(C=CC(=C1)CC=C)O	EUGENOL
COC1=C(C=C(C=C1)CC=C)OC	METHYLEUGENOL

Table S5. Prediction result of deep learning

a. Deep learning prediction result

**SMILES**	**Prediction score (max = 0)**	**Molecule**
Cc1ccc(cc1)C(O)(C)C	0.008643875	GERANIOL+8-HYDROXY-P-CYMENE
C=CC(CCCC(O)(C)C)C	0.002009252	DIHYDROMYRCENOL
CCC(O)C	0.002073357	2-BUTANOL
OCCC(CCCC(C)C)C	0.000890598	3,7-DIMETHYL-1-OCTANOL
C=CC(OC(=O)C)(CCC=C(C)C)C	0.017367085	LINALYL ACETATE
CCC(CO)C	0.00209589	2-METHYL-1-BUTANOL
COc1cc(C)ccc1C(C)C	0.01343793	METHYLTHYMOL
C=CC(CCC=C(C)C)(O)C	0.011044887	LINALOOL
OCCC(CC)C	0.002113451	1-Pentanol, 3-methyl-
OCCC(CCC=C(C)C)C	0.004184846	CITRONELLOL
CC(CCC=C(C)C)CCOC(=O)C	0.009150817	CITRONELLYL ACETATE
CC(=CCCC(c1ccc(cc1)C)C)C	0.029458433	A-CURCUMENE
CC(C1CC=C(c2c1cc(C)cc2)C)C	0.025302203	A-CALACORENE
OCc1ccc(cc1)C(C)C	0.026568791	P-CYMEN-7-OL
Cc1ccc(cc1)C(C)C	0.03990693	P-CYMENE
CCC(=O)OC(CCC=C(C)C)(C=C)C	0.034996018	LINALYL PROPIONATE
CC=C(C(=O)OCCC(CCC=C(C)C)C)C	0.012789104	CITRONELLYL TIGLATE
C=CC(CCC=C(CCC=C(C)C)C)(O)C	0.01516746	NEROLIDOL
Cc1ccc(c(c1)O)C(C)C	0.038171202	THYMOL
CCCCCCC(C)C	0.001651392	2-METHYLOCTANE
CCCCCC(O)C	0.001959076	2-HEPTANOL
O=CCC(CCC=C(C)C)C	0.017464662	CITRONELLAL
Cc1cc(C)c(c(c1C)O)C	0.048647635	2,3,5,6-TETRAMETHYL PHENOL
O=COCCC(CCC=C(C)C)C	0.01350342	CITRONELLYL FORMATE

CCCCCC(CC)O	0.001825806	3-OCTANOL
CC(=CCOC(=O)C)CCC=C(C)C	0.045709711	NERYL ACETATE
OCC=C(CCC=C(C)C)C	0.023227636	NEROL
CCCCCCCC(O)C	0.001822409	2-NONANOL
CCCCC=C(C)C	0.014632422	2-METHYL-2-HEPTENE
CC=C(C(=O)OCC=C(CCC=C(C)C)C)C	0.054050189	GERANYL TIGLATE
C=CC1(C)CCC(O1)C(O)(C)C	0.007967635	LINALOOL OXIDE
OCC=C(CCC=C(CCC=C(C)C)C)C	0.026609171	FARNESOL
O=Cc1ccc(cc1)C(C)C	0.231102541	CUMINAL
CC(C1CCC(c2c1cc(C)cc2)C)C	0.035699572	CALAMENENE
CC(=O)CCC=C(C)C	0.060619555	6-METHYL-5-HEPTEN-2-ONE
Cc1ccc(cc1)C1(C)CCCC1(C)C	0.108623639	(+)CUPARENE
CCCC(=O)OCCC(CCC=C(C)C)C	0.018891722	CITRONELLYL BUTYRATE
CC(=CCCC(=O)C)CCC=C(C)C	0.05185625	GERANYL ACETONE
CCCC=C(C(=O)C)C	0.116623253	METHYL HEPTENONE
O=CC=C(CCC=C(C)C)C	0.139957607	CITRAL
O=CC=C(CCC=C(CCC=C(C)C)C)C	0.093734406	FARNESAL
CC=Cc1ccc(c(c1)OC)OC	0.136565506	METHYL ISOEUGENOL
C=CC1(C)CCC(C(C1C(=C)C)O)C(C)C	0.011093457	6-SHYOBUNOL
CC(C(=O)O)CC	0.130108878	METHYL 2-METHYLBUTYRATE
CCCCCC(C=C)O	0.009655901	1-Octen-3-ol
CC(=CC=CC(=O)C)C	0.414595753	6-METHYL-3,5-HEPTADIEN-2-ONE
CCC(C(=O)OCCc1ccccc1)C	0.33822456	2-PHENYLETHYL 2-METHYLBUTYRATE
CCC(=O)OCC=C(CCC=C(C)C)C	0.101425946	NERYL PROPIONATE
C=CC(=CCC=C(CCC=C(C)C)C)C	0.171454579	A-FARNESENE
CCCC(CC(=O)CCc1ccc(c(c1)OC)O)O	0.069810294	(4)-Gingerol
CCCCC	0.008540599	PENTANE
C=CC(=CCC=C(C)C)C	0.30781278	TRANS-B-OCIMENE
CC(=CCCC(C1CC=C(C=C1)C)C)C	0.03706352	l-Zingiberene
CCCCCC(CC(=O)CCc1ccc(c(c1)OC)O)O	0.044022541	6-Gingerol
C=CCc1ccc(c(c1)OC)OC	0.192272097	METHYLEUGENOL
CCCCCO	0.006227626	1-PENTANOL
CCCCCCCC(CC(=O)CCc1ccc(c(c1)OC)O)O	0.030155448	(8)-Gingerol
O=CCC1CC=C(C1(C)C)C	0.129566833	A-CAMPHOLENAL
CCCC(=O)OCC=C(CCC=C(C)C)C	0.100294523	GERANYL BUTYRATE

CCCCCCCCCC(CC(=O)CCc1ccc(c(c1)OC)O)O	0.022273788	(10)-Gingerol
CCCCCCO	0.005009609	HEXANOL
CC(=O)OCCc1ccccc1	0.482536584	PHENETHYL ACETATE
CC=C(C(=O)OCCc1ccccc1)C	0.390719265	2-Phenylethyl tiglate
CCCCCCCO	0.004298539	HEPTANOL
OCc1ccccc1	0.419901103	BENZYL ALCOHOL
CCCCCCCC	0.004357493	OCTANE
CC1=CCC(C=C1)C(C)C	0.045506459	DIHYDRO P-CYMENE
CCCCCCCCO	0.003805185	OCTANOL
CC(=CCCC(C1(O)CCC(=CC1)C)C)C	0.036208939	B-BISABOLOL
CCCCCC(=O)C	0.027355941	2-HEPTANONE
CCCCCCCCCC	0.003501763	DECANE
CC(=O)OC1C2(C)CCC(C1(C)C)C2	0.060283914	
CC(=CC1OCC=C(C1)C)C	0.059980094	NEROL OXIDE
Cc1ccc(cc1)C(=O)C	0.607619047	P-METHYLACETOPHENONE
OCCc1ccccc1	0.469033599	PHENETHYL ALCOHOL
CCCCCCCC(=O)C	0.015096921	2-NONANONE
CCCCCCCCCCCCC	0.002867836	TRIDECANE
CCOC(=O)c1ccccc1C(=O)OCC	0.301276624	DIETHYL PHTHALATE
CCCC=CCO	0.07663592	TRANS-2-HEXENOL
CCCCCCCCCCCCCCC	0.002627785	PENTADECANE
CCCCCCCCCC(=O)C	0.010120729	METHYL NONYL KETONE
Cc1ccccc1	0.702145159	TOLUENE
CCCCCCCCCCCCCCCC	0.002536088	HEXADECANE
CC(=O)c1ccccc1	0.739649415	ACETOPHENONE
COc1cc(CCC(=O)C)ccc1O	0.323977649	VANILLYLACETONE
CCCCCCCCCCCCCCCCC	0.00245933	HEPTADECANE
OCCC=CCC	0.086930223	LEAF ALCOHOLCC1=CCC(CC1)(C(C)C)O
CCCCCCCCCCCC(=O)C	0.007674633	2-TRIDECANONE
CCCCCCCCCCCCCCCCCC	0.002393398	OCTADECANE
CCCCCCCCCCCCCCCCCCC	0.002335916	NONADECANE
CC(=O)CCc1ccccc1	0.66228646	4-PHENYL-2-BUTANONE
CC(=CCCc1cocc1)C	0.704966903	PERILLEN
CCCCCCCCCCCCCCCCCCCC	0.002285411	EICOSANE
CCCCCCCCCCCCCCCCCCCCC	0.002240672	HENEICOSANE
CC(=O)c1ccc(c(c1)C)O	0.529192209	4-Hydroxy-3-methylacetophenone
CCCCCCCCCCCCCCCCCCCCCCCCCC	0.002076595	HEXACOSANE
CCCCCCCCCCCCCCCCCCCCCCCCCCCC	0.002029751	OCTACOSANE

CC(=O)OC1(C)CCCC(C1=O)(C)C	0.585897207	
CC1=CC(=O)CC(C1)(C)C	0.294976711	ISOPHORONE
C=CC1(C)CCC(CC1C(=C)C)C(O)(C)C	0.165854439	ELEMOL
CCCCCCCCC=CCCCCCCC	0.00929959	8-HEPTADECENE
CCCCCCCCCC=CCCCCCCCC	0.007661625	9-NONADECENE
CCCCCCCCCCC=CCCCCCCCC	0.007051314	9-EICOSENE
CCCCCCCCCCC=CCCCCCCCCC	0.006543405	10-HENEICOSENE
CC(=O)OC1CC2C(C1(C)CC2)(C)C	0.125596374	BORNYL ACETATE
CC1=CC[C@@H](CC1)C(O)(C)C	0.083480217	
CCCCCCOC=O	0.031293157	HEXYL FORMATE
CC(C1CCC(=C)C=C1)CCC=C(C)C	0.174899444	B-sesquiphellandrene
CCCCCC=CC(=O)C	0.197580278	trans-3-Nonen-2-one
C=CCc1ccc(c(c1)OC)O	0.50748986	EUGENOL
CC1=CCC(=CC1)C(C)C	0.213542834	GAMA-TERPINENE
CCCCCCCCCCCCCCCCCC=C	0.004797086	1-NONADECENE
O=C(c1ccccc1)OCc1ccccc1	0.805855095	BENZYL BENZOATE
CC1CCOC(C1)C=C(C)C	0.028964968	ROSE OXIDE
CC1CCC(C(C1)O)C(C)C	0.007774258	ISOMENTHOL
CCCCCCCCCC=O	0.014482718	DECANAL
CCCCCCCCC=O	0.018395383	NONANAL
C=CC(=C)CCC=C(C)C	0.616398215	
CCCCCCCC=O	0.024756061	OCTANAL
C=CC1(C)CCCC(O1)(C)C	0.084495634	LIMTOL
CCCCCCC=O	0.036211517	HEPTANAL
O=C(c1ccccc1)OCCc1ccccc1	0.80821234	PHENETHYL BENZOATE
CCCCCC=O	0.059204407	HEXANAL
CCCC=O	0.230770826	BUTANAL
CCCCC=O	0.107217938	PENTANAL
CC(=O)OC(C1CCC(C2=C(C1)C(C)CC2)C)(C)C	0.182576224	A-TERPINEOL+GUAIYL ACETATE
OC1C2(C)CCC(C1(C)C)C2	0.038045641	Bicyclo[3.1.1]heptan-3-one, 2,6,6-trimethyl-, (1.alpha.,2.beta.,5.alpha.)-
CCCCCC(=O)CC	0.067102708	3-OCTANONE
CC1=CCC(C=C1)C(=C)C	0.421439707	DIHYDRO P-CYMENE
C=CC(CCC1C(C)(O)CCC2C1(C)CCCC2(C)C)(O)C	0.098436795	SCLAREOL
O=Cc1ccccc1	0.950688064	BENZALDEHYDE
C=CC1(C)CCC(=C(C)C)CC1C(	0.550105691	GAMA-ELEMENE

=C)C		
CC1=CC(=O)C2CC1C2(C)C	0.509539425	VERBENONE
CCCCCC=CC(=O)CCc1ccc(c(c1)OC)O	0.486877888	6-Shogaol
CC1C=CC2C(C1)C2(C)C	0.048436388	4-CARENE
CC(C1CCC(=C)C=C1)C	0.430848837	B-PHELLANDRENE
CC(=O)OCC1=CCC2CC1C2(C)C	0.475699037	MYRTENYL ACETATE
OC1CC2C(C1(C)CC2)(C)C	0.111609176	BORNEOL
CC1(C)C2CCC1(C)C=C2	0.217282549	2-BORNENE
CCCCCc1ccco1	0.727123737	2-PENTYLFURAN
CC1=CCC(CC1)C(C)C	0.172558561	MENTHENE
CC1CCC(C(C1)O)C(=C)C	0.052114975	ISOPULEGOL
C=CC(=C)CCCC(=C)C	0.619332373	MYRCENE
CC1=CC2C(CC1)C(=CCC2C(C)C)C	0.082197197	A-MUUROLENE
CC1=CCC(C)(C)C=CCC(=CCC1)C	0.485965103	HUMULENE
CC1=CC2C(=C(C)CCC2C(C)C)CC1	0.120458908	
ON=C1CC(C)CC(C1)(C)C	0.372406363	2,2,6-TRIMETHYLCYCLOHEXANONE
CC1=CC2C(CC1)C(C)(O)CCC2C(C)C	0.057884987	A-CADINOL
CC(=CCCC(=C)C1CCC(=CC1)C)C	0.552127719	B-OCIMENE
CC1=CCC(=C(C)C)CC1	0.608692169	TERPINOLENE
C=C1C(O)CC2CC1C2(C)C	0.213927448	TRANS-PINOCARVEOL
CC1CCC(C(=O)C1)C(C)C	0.322236359	MENTHONE
C=C[C@]1(C)CC[C@H](C[C@H]1C(=C)C)C(=C)C	0.717864037	B-ELEMENE
CC1=CCC2CC1C2(C)C	0.457652032	A-PINENE
CC1=CC=C(CC1)C(=C)C	0.854401529	1,3,8-p-Menthatriene
OCC1=CCC2CC1C2(C)C	0.438805223	MYRTENOL
CC1=CCCC2(C1CC(CC2)C(O)(C)C)C	0.332029194	A-EUDESMOL
CC1=CCC2C(C1)C2(C)C	0.481720418	3-CARENE
CC1CCCC2=CCC3C(C12C)C3(C)C	0.358923465	ARISTOLENE
CC(=CCCC1(C)C2CCC(=C)C1C2)C	0.609347165	BERGAMOTENE
CC1=C2CC(CCC2(CCC1)C)C(O)(C)C	0.456567317	GAMA-EUDESMOL
CC1CCC=C2C1(C)C1C(C1(C)C)CC2	0.408815265	(+)-CALARENE
CC1(O)CC=C2C(CC1)(C)CCCC2(C)C	0.353506804	WIDDROL

CC(C1CCC2(C3C1C2C(=CC3)C)C)C	0.124494724	A-YLANGENE
CC(=C)C1CC=C(C(=O)C1)C	0.894299507	CARVONE
CC(C1CCC(C23C1C2C(=CC3)C)C)C	0.117594391	CUBEBENE
O=CC1=CCC2CC1C2(C)C	0.854127228	MYRTENAL
CC1=CC2C(CC1)C(=C)CCC2C(C)C	0.295259088	GAMA-MUUROLENE
O=C1CC2CC(C1C)C2(C)C	0.76543057	3-PINANONE
CC(=C)C1CCC(CC1)(C)O	0.303873539	P-MENTH-8-EN-1-OL
CC1CCC2C1C1C(C1(C)C)CCC2(C)O	0.113247775	GLOBULOL
C=C1CCC2C(C3C1CCC3(C)O)C2(C)C	0.281699359	SPATHULENOL
CC12CCC(CC1)C(O2)(C)C	0.333440602	EUCALYPTOL
C=C1CCCC2(C1CC(CC2)C(O)(C)C)C	0.61417973	B-EUDESMOL
CC1=CCC(CC1)C(=C)C	0.81270206	LIMONENE
O=C1CC2C(C1(C)CC2)(C)C	0.967295825	CAMPHOR
CC12C3C1CC(C2(C)C)C3	0.513224721	TRICYCLENE
CC1CCC23C1C1C(C1(C)C)CCC3(O2)C	0.220384538	ledene oxide
C=C1CCC2(C1C2)C(C)C	0.90388757	SABINENE
CC(C1CCC2(C3C1C2C(=C)CC3)C)C	0.410767972	B-YLANGENE
CC1=CCC2C(O1)(C)CCC1C2(C)CCCC1(C)C	0.809511542	SCLAREOLOXIDE
C=C1CCC2OC2(CCC2C1CC2(C)C)C	0.479538113	CARYOPHYLLENE OXIDE
CC(C1CCC2(C1C1C(=C)CCC21)C)C	0.454213738	B-BOURBONENE
C=C1C2CCC(C1(C)C)C2	0.877349973	CAMPHENE
CC(=C)C1CCC(C2=C(C1)C(C)CC2)C	0.754229069	A-GUAIENE
CC1=CCCC(=C)C2C(CC1)C(C2)(C)C	0.817911327	B-CARYOPHYLLENE
CC(C1CCC2(C3C1C1C(C21C)C3)C)C	0.227172554	(+)-CYCLOSATIVENE
CC(C1CCC(C23C1C2C(=C)CC3)C)C	0.434811652	
CC1CCC2C1C1C(C1(C)C)CCC2=C	0.478167206	ALLOAROMADENDRENE
C=C1CCC2CC1C2(C)C	0.894185781	B-PINENE
CC(=C)C1CCC(=C2C(C1)C(C)CC2)C	0.762745261	A-BULNESENE
O=C1C2(C)CCC(C1(C)C)C2	0.990963817	FENCHONE
CC1=CCC23CC1C(C)(C)C2CCC3C	0.900035858	(-)-ALPHA-CEDRENE
CC1=CCCC2(C1CC(CC2)C(=C	0.873063326	A-SELINENE

)C)C		
CC1CCC2C31CCC(C(C3)C2(C)C)(C)O	0.855059028	CEDROL
C=C1C2CCC3C1(C)CCCC(C23)(C)C	0.753828704	LONGIFOLENE
CC1=CCC2(C3(C1C3)C(C)(C)CCC2)C	0.96209991	CIS-THUJOPSENE
C=C1CCC(CC1)C(=C)C	0.946295738	p-Mentha-1(7),8-diene

b. 13 hits from LigandScout

**Canonical SMILES**	**Prediction score (max = 0)**	**Molecule**
C=CC1(C)CCC(O1)C(O)(C)C	0.00796764	LINALOOL OXIDE
CCCCCCCCCC(CC(=O)CCc1ccc(c(c1)OC)O)O	0.02227379	(10)-Gingerol
CCCCCCCC(CC(=O)CCc1ccc(c(c1)OC)O)O	0.03015545	(8)-Gingerol
CCCCCC(CC(=O)CCc1ccc(c(c1)OC)O)O	0.04402254	6-Gingerol
CCCC(CC(=O)CCc1ccc(c(c1)OC)O)O	0.06981029	(4)-Gingerol
C=CC(CCC1C(C)(O)CCC2C1(C)CCCC2(C)C)(O)C	0.0984368	SCLAREOL
CC=Cc1ccc(c(c1)OC)OC	0.13656551	METHYL ISOEUGENOL
C=CCc1ccc(c(c1)OC)OC	0.1922721	METHYLEUGENOL
CCOC(=O)c1ccccc1C(=O)OCC	0.30127662	DIETHYL PHTHALATE
COc1cc(CCC(=O)C)ccc1O	0.32397765	Zingerone
CCCCCC=CC(=O)CCc1ccc(c(c1)OC)O	0.48687789	6-Shogaol
C=CCc1ccc(c(c1)OC)O	0.50748986	EUGENOL
CC(=O)c1ccc(c(c1)C)O	0.52919221	4-Hydroxy-3-methylacetophenone

Table S6. Calcium channel blockers used to train the deep neural network

CID	Chemical Name	MW
656667	Benidipine hydrochloride	542
44146578	(-)-alpha-Benidipine hydrochloride	542
656668	Benidipine	505.6
10029174	(3S,4'R)-Benidipine HCl	505.6
102601872	(-)-Alfa-Benidipine HCl	542
5749098	(3R,4'S)-Benidipine HCl	505.6
71313656	Rac Benidipine-d5 Hydrochloride	547.1
71776387	(+)-Alfa-Benidipine HCl	542
123768	BENIDIPINE HCl	542
90478234	Rac-Benidipine HCl	505.6

3088702	3,5-Pyridinedicarboxylic acid, 1,4-dihydro-2,6-dimethyl-4-(3-nitrophenyl)-, methyl 1-(phenylmethyl)-3-piperidinyl ester, monohydrochloride, (S-(R*,R*))-	542
134694739	5-O-(1-Benzylpiperidin-3-yl) 3-O-methyl (4R)-2,6-dimethyl-4-(3-nitrophenyl)-1,4-dihydropyridine-3,5-dicarboxylate;hydrochloride	542
101526732	[(4R)-5-[(3R)-1-Benzylpiperidin-1-ium-3-yl]oxycarbonyl-2,6-dimethyl-4-(3-nitrophenyl)-4H-pyridin-3-ylidene]-methoxymethanolate	505.6
62920	Diltiazem hydrochloride	451
39186	Diltiazem	414.5
3037122	Diltiazem malate	548.6
198107	L-cis-Diltiazem	414.5
3076	Dilacor XR	414.5
101117241	Diltiazem Sulfoxide	430.5
45039052	Diltiazem-d3 HCl	454
67938926	Diltiazem maleate	530.6
129734791	Diltiazem-carbamazepine	650.8
54400293	Diltiazem N-oxide	430.5
71315733	N-Desmethyl Diltiazem-d4 Hydrochloride	441
3035956	3-(4-Azidobutyryloxy)diltiazem	483.6
45038852	Desacetyl Diltiazem-d3	375.5
51057199	Diltiazem 3 Methyl Lactum	315.4
71315571	Desacetyl Diltiazem-d6	378.5
71315569	Desacetyl Diltiazem-d4	376.5
131700451	Deacetyl-O-demethyl Diltiazem Hydrochloride	394.9
49849632	Diltiazem-d4 Hydrochloride	455
67827136	US9138393, Diltiazem	414.5
145714390	Des[5-(2-dimethylamino)ethyl] N-Methyl Diltiazem	357.4
139201529	Diltiazem l-malate	548.6
131770006	N-Desmethyl Diltiazem	400.5
122199558	N,N,O-Tridesmethyl Diltiazem	372.4
3035520	3-(4-Azidobenzoyloxy)diltiazem	517.6
11068728	Desacetyl Diltiazem	372.5
67937026	Diltiazem hydroiodide	542.4
122199468	O-Desmethyl Diltiazem Hydrochloride	437
91810616	N-Desmethyl desacetyl diltiazem hydrochloride	394.9
91748344	Diltiazem, M(O-desmethyl-desamino-HO-), AC	457.5
978317	d-trans-Diltiazem	414.5
6957965	Diltiazem(1+)	415.5
10804253	Diltiazem trans-(-)-Hydroxy Lactam	301.4
23620717	(+)-trans-Diltiazem hydrochloride	487.4
45039053	Diltiazem-d3	417.5

71315709	Diltiazem, M(odesmethyl-), AC	442.5
71316027	N,N-Didesmethyl Diltiazem-d4 Hydrochloride	427
75125723	Desacetyl Diltiazem-d6 hydrochloride	415
131732427	Diltiazem Eudragit Triethly Citrate	790.9
122236667	Desacetyl Diltiazem Hydrochloride (50 mg)	408.9
6957646	Ent-diltiazem(1+)	415.5
13100698	Diltiazem Impurity SA19520	333.4
18601406	(2R-trans)-Diltiazem Hydrochloride	451
45038878	N-Desmethyl Diltiazem-d3 Hydrochloride	440
71315210	Deacetyl Diltiazem N-Oxide	388.5
71316026	N,N-Didesmethyl Diltiazem Hydrochloride	422.9
71433758	O-Desmethyl Diltiazem	437
75125722	Diltiazem-d6 hydrochloride	457
126456094	Deacetyl-O-demethyl Diltiazem Hydrobromide	439.4
14391644	N-Demethyldiltiazem hydrochloride	437
158945	1,5-Benzothiazepin-4(5H)-one, 2,3-dihydro-3-hydroxy-2-(4-methoxyphenyl)-5-(2-(methylamino)ethyl)-, (2S,3S)-	358.5
198106	(2R-cis)-3-Acetoxy-5-(2-(dimethylamino)ethyl)-2,3-dihydro-2-(4-methoxyphenyl)-1,5-benzothiazepin-4(5H)-one monohydrochloride	451
91638	Deacetyldiltiazem	372.5
2733673	(2S,3s)-3-Hydroxy-2-(4-methoxyphenyl)-2,3-dihydro-1,5-benzothiazepin-4(5h)-one	301.4
11110671	(2S)-cis-3-(Acetyloxy)-2,3-dihydro-2-(4-methoxyphenyl)-1,5-benzothiazepin-4(5H)-one	343.4
107891	N-Demethyldiltiazem	400.5
10021324	O-Demethyldeacetyldiltiazem	358.5
124580970	(2S,3S)-5-(2-Aminoethyl)-3-hydroxy-2-(4-hydroxyphenyl)-2,3-dihydro-1,5-benzothiazepin-4-one	330.4
124929793	2-[(2R,3R)-3-Hydroxy-2-(4-methoxyphenyl)-4-oxo-2,3-dihydro-1,5-benzothiazepin-5-yl]-N,N-dimethylethanamine oxide	388.5
91810591	O-Demethyldeacetyldiltiazem hydrochloride	394.9
10472681	Deacetyl-N,O-didemethyldiltiazem	344.4
214464	3-Acetoxy-5-[2-(dimethylamino)ethyl]-2,3-dihydro-2-(4-methoxyphenyl)-1,5-benzothiazepin-4(5H)-one monohydrochloride	451
12449426	Deacetyldiltiazem hydrochloride	408.9
71315570	(2S,3S)-3-Hydroxy-2-(4-methoxyphenyl)-5-[2-[methyl(trideuteriomethyl)amino]ethyl]-2,3-dihydro-1,5-benzothiazepin-4-one	375.5
75125720	[(2S,3S)-2-(4-Methoxyphenyl)-5-[2-(methylamino)ethyl]-4-oxo-2,3-dihydro-1,5-benzothiazepin-3-yl] 2,2,2-trideuterioacetate;hydrochloride	440

218444	1,5-Benzothiazepin-4(5H)-one, 2,3-dihydro-5-(2-(dimethylamino)ethyl)-3-hydroxy-2-(p-hydroxphenyl)-, monohydrochloride	394.9
131632891	[(2R,3R)-5-[2-[Bis(trideuteriomethyl)amino]ethyl]-2-(4-methoxyphenyl)-4-oxo-2,3-dihydro-1,5-benzothiazepin-3-yl] acetate;hydrochloride	457
978318	[(2~{S},3~{R})-5-[2-(Dimethylamino)ethyl]-2-(4-methoxyphenyl)-4-oxidanylidene-2,3-dihydro-1,5-benzothiazepin-3-yl] ethanoate	414.5
218442	1,5-Benzothiazepin-4(5H)-one, 2,3-dihydro-5-(2-(dimethylamino)ethyl)-3-hydroxy-2-(p-methoxyphenyl)-, monohydrochloride, (Z)-	408.9
5282407	Flunarizine hydrochloride	477.4
941361	Flunarizine	404.5
129318432	Flunarizine N-Oxide Dihydrochloride	493.4
35353	Flunarizine * 2HCl	477.4
6438877	Hydroxy Flunarizine	420.5
14386607	Flunarizine impurity B	386.5
129318431	4-Defluoro 2-Fluoro Flunarizine	404.5
1711971	1-[Bis(4-fluorophenyl)methyl]-4-[(Z)-3-phenyl-2-propenyl]piperazine	404.5
3784	Isradipine	371.4
10406778	Dehydro Isradipine	369.4
29976815	Dehydro Isradipine Lactone	353.3
45039616	Isradipine-d3	374.4
46782005	Isradipine Lactone	355.3
20161387	Demethyl Propionitrile Isradipine	410.4
158617	3,5-Pyridinedicarboxylic acid, 4-(2,1,3-benzoxadiazol-4-yl)-1,4-dihydro-2,6-dimethyl-, methyl 1-methylethyl ester, (4S)-	371.4
14519595	4-(2,1,3-Benzoxadiazol-4-yl)-1,4-dihydro-2,6-dimethyl-3,5-pyridinedicarboxylic acid, 3-(1-methylethyl) ester	357.4
41114	Nicardipine hydrochloride	516
4474	Nicardipine	479.5
3017161	Nicardipine pyridine metabolite II	477.5
6604415	(S)-Nicardipine	479.5
56841549	Moexipril mixture with nicardipine	1014.6
6603948	(R)-Nicardipine	479.5
66587092	(S)-Nicardipine hydrochloride	516
71750994	Nicardipine D3 (hydrochloride)	519
73416090	(R)-Nicardipine hydrochloride	516
529921	Nicardipine M (dehydro-desamino-carboxy, methyl ester)	402.4
129318587	Nicardipine N-Oxide	495.5
12609737	Dehydro Nicardipine Hydrochloride	514

529922	Nicardipine M (dehydro-desamino-hydroxy)	374.3
177986	Nicardipine phosphate	577.5
45040010	Nicardipine-d3	482.5
12529164	De-benzylated nicardipine	389.4
529924	Dimethyl 2,6-dimethyl-4-(3-nitrophenyl)pyridine-3,5-dicarboxylate	344.32
173322	5-(Methoxycarbonyl)-2,6-dimethyl-4-(3-nitrophenyl)-1,4-dihydropyridine-3-carboxylic acid	332.31
4485	Nifedipine	346.3
15364971	Nifedipine pyrimidine	398.4
45040023	Nifedipine D6	352.37
13599653	Hydroxydehydro Nifedipine Carboxylate	346.29
13599652	Hydroxy Dehydro Nifedipine Lactone	328.28
63011	Nifedipine hydrochloride	382.8
89082	M-Nifedipine	346.3
10592508	Desmethyl hydroxymethyl nifedipine lactone	330.29
45038793	Dehydro Nifedipine-d6	350.35
49849384	[13C8]-Oxidized nifedipine	352.26
49849382	Nifedipine-13C8, 99 atom % 13C	354.28
86742527	Nifedipine isothiocyanate	404.4
129762646	Dihydropyridyl nifedipine	425.4
129669919	Bromo-nifedipine	425.2
529925	Nifedipine M (dehydro-bis-carboxy, methyl ester)	388.3
129780964	o-(Trifluoro-methyl)phenyl nifedipine	490.4
129693714	Cisplatin nifedipine	526.4
129669923	4-Nitro nifedipine	391.3
129669924	4-Bromo nifedipine	425.2
129724666	Nifedipine lactate	434.4
128753	Dimethyl 2,6-dimethyl-4-(2-nitrophenyl)-3,5-pyridinedicarboxylate	344.32
128034	Dimethyl 2,6-dimethyl-4-(2-nitrosophenyl)pyridine-3,5-dicarboxylate	328.32
173322	5-(Methoxycarbonyl)-2,6-dimethyl-4-(3-nitrophenyl)-1,4-dihydropyridine-3-carboxylic acid	332.31
54167661	2,6-Dimethyl-4-(2-nitrophenyl)-3,4-dihydropyridine-3,5-dicarboxylic acid dimethyl ester	346.3
486145	(3S,6S,9S,12R,15R,18S,21S,24S,27S)-3,6-Dibenzyl-15-(1-hydroxy-1-methyl-ethyl)-18-isobutyl-9,21-diisopropyl-4,10,16,22-tetramethyl-24-[(1S)-1-methylpropyl]-12-[(1R)-1-methylpropyl]-13-oxa-1,4,7,10,16,19,22,25-octazabicyclo[25.3.0]triacontane-2,5,8,11,14,17,20,23,26-nonone; dimethyl 2,6-dimethyl-4-(2-nitrophenyl)-1,4-dihydropyridine-3,5-dicarboxylate	1447.8
461539	1-[4-[4-[[(2R,4S)-2-(2,4-Dichlorophenyl)-2-(imidazol-	877.8

	1-ylmethyl)-1,3-dioxolan-4-yl]methoxy]phenyl]piperazin-1-yl]ethanone; dimethyl 2,6-dimethyl-4-(2-nitrophenyl)-1,4-dihydropyridine-3,5-dicarboxylate	
152434	Dimethyl 2,6-dimethyl-4-(4-nitrophenyl)-1,4-dihydropyridine-3,5-dicarboxylate	346.3
489226	Dimethyl 2,6-dimethyl-4-(2-nitrophenyl)-1,4-dihydropyridine-3,5-dicarboxylate; 2-(2-methyl-5-nitro-imidazol-1-yl)ethanol	517.5
4497	Nimodipine	418.4
529928	Dehydro nimodipine	416.4
101749934	Monohydroxy of nimodipine	434.4
15953942	Ketoconazole & Nimodipine	949.9
529929	Nimodipine M (dehydro-desisopropyl-O-desmethyl, methyl ester)	388.4
529930	Nimodipine M (dehydro-desmethoxyethyl, methyl ester)	372.4
91748590	Nimodipine M (dehydro-O-desmethyl-carboxy, methyl ester)	416.4
157132	3,5-Pyridinedicarboxylic acid, 1,4-dihydro-2,6-dimethyl-4-(3-nitrophenyl)-, 2-methoxyethyl 1-methylethyl ester, (4R)-	418.4
157133	3,5-Pyridinedicarboxylic acid, 1,4-dihydro-2,6-dimethyl-4-(3-nitrophenyl)-, 2-methoxyethyl 1-methylethyl ester, (4S)-	418.4
10431202	Unii-96S4GG1upr	402.4
13219259	2-Hydroxyethyl 2-methyl-4-(3-nitrophenyl)-5-oxo-5,7-dihydrofuro(3,4-b)pyridine-3-carboxylate	358.3
10453860	2-Hydroxyethyl isopropyl 1,4-dihydro-2,6-dimethyl-4-(3-nitrophenyl)pyridine-3,5-dicarboxylate	404.4
529921	Nicardipine M (dehydro-desamino-carboxy, methyl ester)	402.4
529924	Dimethyl 2,6-dimethyl-4-(3-nitrophenyl)pyridine-3,5-dicarboxylate	344.32
2520	Verapamil	454.6
62969	Verapamil hydrochloride	491.1
12249889	(-)-Verapamil Hydrochloride	491.1
65808	Dexverapamil	454.6
92305	(-)-Verapamil	454.6
24847846	Trandolapril and Verapamil Hydrochloride	921.6
86585500	Verapamil verapamil hydrochloride	945.7
16220090	(R)-(+)-Verapamil (hydrochloride)	509.1
627485	Desisopropyl N-propyl verapamil	454.6
137314795	Verapamil & Rifampicin	1277.5
5312288	[11C]-Verapamil	453.6
127053753	Bodipy-verapamil	732.7
21124838	R-Verapamil HCl	477
45359134	Nor Verapamil-d7, Hydrochloride	484.1

134814874	Verapamil & Amikacin	1040.2
134814746	Verapamil & Ethidium Bromide	769
71596372	TDR58845 with verapamil	704.3
134815124	Verapamil & Ofloxacin	816
157039	p-O-Desmethyl Verapamil	440.6
57358013	Desisopropyl ethyl verapamil	440.6
71596237	TDR58846 with verapamil	732.4
134815085	Verapamil & Isoniazid	591.7
46783239	Verapamil Ethyl Methanethiosulfonate, Bromide	673.7
492231	Trifluralin & Verapamil	789.9
69842939	Verapamil-nitrate	517.6
71315157	D 517-d7 Hydrochloride (Verapamil Impurity)	484.1
129722827	Verapamil vanadate	569.5
129712053	Verapamil alcohol	470.6
101529637	Nor Verapamil N-|A-D-Glucuronide	616.7
45359132	Verapamil-D7 Hydrochloride	498.1
46781152	p-O-Desmethyl p-O-Benzyl Verapamil	530.7
71312145	S(-)-Verapamil hydrochloride hydrate, >=98% (HPLC), powder	509.1
129861714	Verapamil tartrate	600.7
129675778	Azido-verapamil	495.6
126456044	(S)-(-)-Verapamil-d6 Hydrochloride	497.1
146681202	(R)-Verapamil D7 (hydrochloride)	498.1
6429774	Verapamil, M(nor-), AC	482.6
46783238	Verapamil-d6 Hydrochloride	497.1
129773950	Verapamil rhodamine	556.5
129777606	Acetyl verapamil	496.6
129646102	Verapamil chloride	489
5748292	Nab-verapamil	607.8
135121636	Verapamil hydrate	472.6
57323485	N-Desmethyl Verapamil-d7 HCl	267.4
486141	Aureobasidin A & Verapamil	1556
129667933	Carboxy verapamil	498.6
170014	Dexverapamil Hydrochloride	491.1
93168	3-(3,4-Dimethoxyphenyl)-2-methyl-6-methylaminohexane-3-carbonitrile	290.4
71315155	D-517 Hydrochloride	477
8419	Veratraldehyde	166.17
7118	3,4-Dimethoxybenzyl alcohol	168.19
104972	Norverapamil	440.6
155002	Norverapamil hydrochloride	477
129318328	5,5'-(Methylimino)bis(2-(3,4-dimethoxyphenyl)-2-(1-methylethyl)pentanenitrile)	549.7
15593907	Arverapamil	440.6

72941683	N,N'-Bis(2-(3,4-dimethoxyphenyl)ethyl)-N,N'-dimethylpropane-1,3-diamine	430.6
6603929	(S)-Gallopamil	484.6
10863369	(2R)-2-(3,4-Dimethoxyphenyl)-5-[2-(3,4-dimethoxyphenyl)ethyl-methylamino]-2-propan-2-ylpentanenitrile	453.6
12883724	(S)-Gallopamil hydrochloride	521.1
16205097	[7-Cyano-1,7-bis(3,4-dimethoxyphenyl)nonan-3-yl]ammonium chloride	477
461538	1-[4-[4-[[(2R,4S)-2-(2,4-Dichlorophenyl)-2-(imidazol-1-ylmethyl)-1,3-dioxolan-4-yl]methoxy]phenyl]piperazin-1-yl]ethanone; 2-(3,4-dimethoxyphenyl)-5-[2-(3,4-dimethoxyphenyl)ethyl-methyl-amino]-2-isopropyl-pentanenitrile	986
355506	N,N-Dimethylhomoveratrylamine	209.28
355505	2-(3,4-Dimethoxyphenyl)-N,N-dimethylethanamine hydrochloride	245.74
56842608	CID 56842608	477
137552081	2,6-Bis(3,4-dimethoxyphenyl)-2,6-bis(1-methylethyl)-heptane-1,7-dinitrile	478.6
7048574	Dexverapamil(1+)	455.6
71308442	(R)-Gallopamil hydrochloride	521.1
86579834	(2S,3R,4R)-4-(6-Bromo-2-methoxy-3-quinolyl)-1-(dimethylamino)-3-(1-naphthyl)-4-phenyl-butan-2-ol; (2S)-2-(3,4-dimethoxyphenyl)-5-[2-(3,4-dimethoxyphenyl)ethyl-methyl-amino]-2-isopropyl-pentanenitrile; (2R)-2-(3,4-dimethoxyphenyl)-5-[2-(3,4-dimethoxyphenyl)ethyl-methyl-amino]-2-isopropyl-pentanenitrile	1464.7
197910	3,4-Dimethoxy-N-methylphenethylamine hydrochloride	231.72
71315156	(2RS)-2-(3,4-Dimethoxyphenyl)-2-(2-((2-(3,4-dimethoxy-phenyl)ethyl)(methyl)amino)ethyl)-3-methylbutanenitrile	440.6
72941684	5,5'-((2-(3,4-Dimethoxyphenyl)ethyl)imino)bis(2-(3,4-dimethoxyphenyl)-2-(1-methylethyl)pentanenitrile)	699.9
89396	2-(3,4-Dimethoxyphenyl)-3-methylbutanenitrile	219.28
577693	N-Methyl-N-(3-chloropropyl)homoveratrylamine	271.78

Supplementary Table 7. Plasmids and oligonucleotides designed and used in this study

Plasmid	Description and Cloning Strategy	Reference or source
pCa$_V$1.2	pcDNA3.1(+)-derived constitutive Cacna1c expression vector (P_{hCMV}-Cacna1c-pA) (Addgene no. 26572).	(Helton et al., 2005)
pCa$_V$1.3	pcDNA3.1(+)-derived constitutive Cacna1d expression vector (P_{hCMV}-Cacna1d-pA) (Addgene no. 49333).	(Xu and Lipscombe, 2001)
pCa$_V$1.3:Δ42a	pcDNA3.1(+)-derived constitutive Cacna1d (exon 42a was deleted) expression vector (P_{hCMV}-Cacna1d:Δ42a-pA) (Addgene no. 49332).	(Xu and Lipscombe, 2001)
pCav2.2	pcDNA3.1(+)-derived constitutive Cacna1b expression vector (P_{hCMV}-Cacna1b-pA) (Addgene no. 26569).	(Bell et al., 2004)
pCavb3	pcDNA3.1(+)-derived constitutive Cacnb3 expression vector (P_{hCMV}-Cacnb3-pA) (Addgene no. 26574).	(Lin et al., 2004)
pCaVα2δ1	pcDNA3.1(+)-derived constitutive Cacna2d1 expression vector (P_{hCMV}-Cacna2d1-pA) (Addgene no. 26575).	(Lin et al., 2004)
pcDNA3.1(+)	Mammalian expression vector (P_{hCMV}-MCS-pA).	Invitrogen, CA
pDsRed-Express-DR	Promoter-less vector encoding DsRed-Express (DsRed-Express-pA).	Clontech, CA
pFS119	P_{NFAT3}-driven TurboGFP:dest1 expression vector (P_{NFAT3}-TurboGFP:dest1-pA; P_{NFAT3}, $(NFAT_{IL4})_5$-P_{min}).	(Xie et al., 2016)
pHY30	P_{NFAT1}-driven SEAP expression vector (P_{NFAT1}-SEAP-pA; P_{NFAT1}, $(NFAT_{IL2})_3$-P_{min}).	(Ye et al., 2011)

pKK56	Constitutive Cacna2d1 and Cacnb3 expression vector ($P_{hEF1\alpha}$-Cacna2d1-P2A-Cacnb3-pA).	(Xie et al., 2016)
pMM54	Tetracycline-responsive d2YFP expression vector ($P_{hCMV*-1}$-d2YFP-pA).	(Muller et al., 2017)
pMX57	P_{NFAT3}-driven SEAP expression vector (P_{NFAT3}-SEAP-pA; P_{NFAT3}, $(NFAT_{IL4})_5$-P_{min}).	(Xie et al., 2016)
pMX58	P_{NFAT4}-driven SEAP expression vector (P_{NFAT4}-SEAP-pA; P_{NFAT4}, $(NFAT_{IL4})_7$-P_{min}).	(Xie et al., 2016)
pMX59	P_{NFAT5}-driven SEAP expression vector (P_{NFAT5}-SEAP-pA; P_{NFAT5}, $(NFAT_{IL4})_9$-P_{min}).	(Xie et al., 2016)
pMX109	P_{NFAT3}-driven PMS expression vector (P_{NFAT3}-PMS-pA).	(Wang et al., 2018)
pSA91	Erythromycin-responsive L7Ae expression vector (P_{ETR2}-L7Ae-pA).	(Auslander et al., 2012)
pSEAP2-Control	Constitutive SEAP expression vector (P_{SV40}-SEAP-pA).	Clontech, CA
pSP16	P_{CREm}-driven SEAP expression vector (P_{CREm}-SEAP-pA).	(Saxena et al., 2016)
pWH5	Paraben-inducible SEAP expression vector (P_{PMS}-SEAP-pA).	(Wang et al., 2015)
pWH9	Constitutive PMS expression vector (P_{SV40}-PMS-pA).	(Wang et al., 2015)
pWH17	Constitutive PMS expression vector (P_{hCMV}-PMS-pA).	(Wang et al., 2018)
pWH29	P_{NFAT3}-driven GLuc expression vector (P_{NFAT3}-GLuc-pA).	(Xie et al.,

		2016)
pWH90	P_{CRE2}-driven SEAP-expression vector (P_{CRE2}-SEAP-pA).	(Wang et al., 2018)
pMX252	SB100X-specific transposon containing a constitutive BFP and PuroR expression unit and a constitutive Cacna1d expression unit (ITR-$P_{hEF1\alpha}$-Cacna1d-pA:P_{RPBSA}-BFP-P2A-PuroR-pA-ITR).	(Xie et al., 2016)
pViM41	Prokaryotic mCherry expression vector (P_{T7}-mCherry-MCS).	Viviani et al., unpublished
pMX89	Constitutive DsRed-Express expression vector (P_{SV40}-DsRed-Express-pA).<br>DsRed-Express was PCR-amplified from pDsRed-Express-DR using oligonucleotides OMX97 (5'-caacagaattccaccATGGCCTCCTCCGAGGACGTCATCAAGGAG-3') and OMX98 (5'-gatcctctagattaCTACACATTGATCCTAGCAGAAGCAC-3'), restricted with *Eco*RI/*Xba*I and cloned into the corresponding sites of pSEAP2-Control.	This work
pMX112	P_{NFAT5}-driven PMS expression vector (P_{NFAT5}-PMS-pA).<br>P_{NFAT5} was excised from pMX59 using *Mlu*I/*Eco*RI and ligated into the corresponding sites (*Mlu*I/*Eco*RI) of pWH17.	This work
pMX124	P_{NFAT3}-driven L7Ae expression vector (P_{NFAT3}-L7Ae-pA).<br>L7Ae was PCR-amplified from pSA91 using oligonucleotides OMX188 (5'-gttggtaaagaattctgcagtcgacggtaccgcgggcccgggatccaccggtcgCCACCATGTACGTGCGCTTCGAGGTGCCCG-3') and OMX189 (5'-catgtctgctcgaagcggccggccgccccgactctagagtcgcggccgcgcatTCACTTCTGCAGGCCCTTGATCTTTTCCAC-3'), restricted with *Eco*RI/*Fse*I and cloned into the corresponding sites (*Eco*RI/*Fse*I) of pWH34.	This work
pMX125	P_{NFAT4}-driven L7Ae expression vector (P_{NFAT4}-L7Ae-pA).<br>L7Ae was PCR-amplified from pSA91 using oligonucleotides OMX188 (5'-gttggtaaagaattctgcagtcgacggtaccgcgggcccgggatccaccggtcgCCACCATGTACGTGCGCTTCGAGGTGCCCG-3') and OMX189 (5'-catgtctgctcgaagcggccggccgccccgactctagagtcgcggccgcgcatTCACTTCTGCAGGCCCTTGATCTTTTCCAC-3'), restricted with *Eco*RI/*Fse*I and cloned into the corresponding sites (*Eco*RI/*Fse*I) of pWH35.	This work

pMX126	P_{NFAT5}-driven L7Ae expression vector (P_{NFAT5}-L7Ae-pA).<br>L7Ae was PCR-amplified from pSA91 using oligonucleotides OMX188 (5'-gttggtaaagaattctgcagtcgacggtaccgcgggcccgggatccaccggtcgCCACCATGTACGTGCGCTTCGAGGTGCCCG-3') and OMX189 (5'-catgtctgctcgaagcggccggccgccccgactctagagtcgcggccgcgcatTCACTTCTGCAGGCCCTTGATCTTTTCCAC-3'), restricted with *Eco*RI/*Fse*I and cloned into the corresponding sites (*Eco*RI/*Fse*I) of pWH36.	This work
pMX195	L7Ae-repressible SEAP expression vector (P_{SV40}-(C/Dbox)$_1$-SEAP-pA).<br>SEAP was PCR-amplified from pWH5 using oligonucleotides OMX195 (5'-cttttgcaaaaagcttcaccgggcgtgatccgaaaggtgacccggtggaattcgcCCACCATGCTGCTGCTGCTGCTGCTGCTGGG-3') and OMX24 (5'-CTTGAGCACATAGCCTGGACCGTTTCCGTA-3'), restricted with *Hind*III/*Nde*I and cloned into the corresponding sites (*Hind*III/*Nde*I) of pWH5.	This work
pMX199	L7Ae-repressible SEAP expression vector (P_{SV40}-(C/Dbox)$_2$-SEAP-pA).<br>SEAP was PCR-amplified from pWH5 using oligonucleotides OMX199 (5'-gcaaaaagcttcaccgggcgtgatccgaaaggtgacccggtgagagacgaccgaagggcgtgatccgaaaggtgacccttcgggaattcgcCCACCATGCTGCTGCTGCTGCTGCTGCTG -3') and OMX24 (5'-CTTGAGCACATAGCCTGGACCGTTTCCGTA-3'), restricted with *Hind*III/*Nde*I and cloned into the corresponding sites (*Hind*III/*Nde*I) of pWH5.	This work
pWH17	Constitutive PMS expression vector (P_{hCMV}-PMS-pA).<br>PMS was excised from pWH09 using *Not*I/*Xba*I and ligated into the corresponding sites (*Not*I/*Xba*I) of pcDNA3.1(+).	This work
pWH31	P_{cFOS}-driven SEAP expression vector (P_{cFOS}-SEAP-pA; P_{cFOS}, (c-fos)$_3$-P_{min}).<br>OMX123 (5'-ctagCAGCCTGACGTTTCAGAGACTGACGTTTCAGAGACTGACGTTTCAGATCTC-3') and OMX126 (5'-tcgaGAGATCTGAAACGTCAGTCTCTGAAACGTCAGTCTCTGAAACGTCAGGCTG-3') were annealed and cloned into the *Xho*I/*Nhe*I-restricted pSP16.	This work
pWH34	P_{NFAT3}-driven d2YFP expression vector (P_{NFAT3}-d2YFP-pA).<br>P_{NFAT3} was excised from pMX57 using *Mlu*I/*Eco*RI and ligated into the corresponding sites (*Mlu*I/*Eco*RI) of pMM54.	This work

pWH35	P_{NFAT4}-driven d2YFP expression vector (P_{NFAT4}-d2YFP-pA).<br>P_{NFAT4} was excised from pMX58 using *Mlu*I/*Eco*RI and ligated into the corresponding sites (*MluI*I/*Eco*RI) of pMM54.	This work
pWH36	P_{NFAT5}-driven d2YFP expression vector (P_{NFAT5}-d2YFP-pA).<br>P_{NFAT5} was excised from pMX59 using *Mlu*I/*Eco*RI and ligated into the corresponding sites (*MluI*I/*Eco*RI) of pMM54.	This work
pWH37	P_{NFAT3}-driven DsRed-Express expression vector (P_{NFAT3}-DsRed-Express-pA).<br>P_{NFAT3} was excised from pMX57 using *Mlu*I/*Eco*RI and ligated into the corresponding sites (*MluI*I/*Eco*RI) of pMX89.	This work
pWH75	P_{NFAT4}-driven PMS expression vector (P_{NFAT4}-PMS-pA).<br>P_{NFAT4} was excised from pMX58 using *Nhe*I/*Eco*RI and ligated into the compatible sites (*Spe*I/*Eco*RI) of pWH17.	This work
pWH135	L7Ae-repressible SEAP expression vector (P_{SV40}-$(C/Dbox)_4$-SEAP-pA).<br>Custom-designed $(C/Dbox)_4$ synthesized from gBlocks® was digested with *Hin*dIII/*Eco*RI, and ligated into the corresponding sites (*Hin*dIII/*Eco*RI) of pSEAP2-control.	This work
pWH145	Constitutive L7Ae expression vector (P_{hCMV}-L7Ae-pA).<br>L7Ae was excised from pSA91 using *Nhe*I/*Xba*I and ligated into the compatible sites (*Nhe*I/*Xba*I) of pcDNA3.1(+).	This work
pWH154	Constitutive Cav1.3^{Y1048A} expression vector (P_{hCMV}- Cacna1d^{Y1048A}-pA).<br>Fragment 1 was PCR-amplified from pMX252 using oligonucleotides OLYL301(5'-ggagacccaagctggctagcgccaccATGCAGCATCAACG-3') and OLYL302 (5'-ATGATGATggcGATAATGAAGAAGATGGAGATCTCCACACG-3'), Fragment 2 was PCR-amplified from pMX252 using oligonucleotides OLYL303 (5'-TTCATTATCgccATCATCATCGTGGCCTTCTTCATG-3') and OLYL304(5'-gctggatatctgcagaattcCTCATCAGAGCATCCGTTCAAGCATC-3'), both fragments were cloned into the corresponding sites (*Nhe*I/*Eco*RI) of pcDNA3.1(+) by homologous recombination using the ClonExpress® MultiS One Step Cloning Kit.	This work

pWH155	Constitutive Cav1.3$^{Y1365A, A1369S, I1372A}$ expression vector (P_{hCMV}- Cacna1d$^{Y1365A, A1369S, I1372A}$-pA). Fragment 1 was PCR-amplified from pMX252 using oligonucleotides OLYL301(5'-ggagacccaagctggctagcgccaccATGCAGCATCAACG-3') and OLYL305 (5'-gcCAGGAAgctACAGAGCATGgcAAAGCTGATGAAGTAGACAATGGC-3'), Fragment 2 was PCR-amplified from pMX252 using oligonucleotides OLYL306 (5'-gcCATGCTCTGTagcTTCCTGgcCATCAACCTCTTCGTGGCTGTCATC-3') and OLYL304(5'-gctggatatctgcagaattcCTCATCAGAGCATCCGTTCAAGCATC-3'), both fragments were cloned into the corresponding sites (*Nhe*I/*Eco*RI) of pcDNA3.1(+) by homologous recombination using the ClonExpress® MultiS One Step Cloning Kit.	This work

Oligonucleotides: Restriction endonuclease-recognition sites are underlined and annealing nucleotides are shown in capital letters.

Abbreviations: **BFP**, blue fluorescent protein; **Ca_V1.2**, member 2 of the Ca_V1 family of L-type voltage-gated Ca^{2+} channels; **Cacna1c**, α1-subunit of mouse Ca_V1.2 (NCBI Gene ID: 12288); **Cav1.3**, member 3 of the Cav1 family of L-type voltage-gated Ca^{2+} channels; **Cav1.3^{AXB}**, synthetic Cav1.3 mutant in which amino acid A at position X of the α1-subunit is replaced with amino acid B; **Cav2.2**, N-type voltage-gated Ca^{2+} channel; **Cacna1d**, α1-subunit of rat Cav1.3 (NCBI Gene ID: 29716); **Cacna1d ^{AXB}**, mutant α1-subunit of Cav1.3 in which amino acid A at position X is replaced with amino acid B; **Cacna1d:Δ42a**, Cacna1d-isoform lacking exon 42; **Cacna1b,** α1-subunit of rat Cav2.2 (NCBI Gene ID: 257648); **Cacnb3**, β3-subunit of rat Cav1.3 (NCBI Gene ID: 25297); **Cacna2d1**, α2δ-subunit of rat Cav1.3 (NCBI Gene ID: 25399); **C/D-box,** L7Ae-specific RNA aptamer (sequence: 5'-GGGCGUGAUCCGAAAGGUGACCC-3'); **c-fos**, human proto-oncogene from the Fos family of transcription factors; **CRE**, cAMP-response element (CREB1 binding site); **d2YFP,** destabilized variant of yellow fluorescent protein; **DsRed-Express,** destabilized variant of *Discosoma sp*. red fluorescent protein; **GLuc**, *Gaussia princeps* luciferase; **IL4**, murine interleukin 4; **KRAB**, Krueppel-associated box protein of the human kox-1 gene; **L7Ae**, archaeal ribosomal protein; **MCS**, multiple cloning site; **NFAT**, nuclear factor of activated T-cells; **O_{PmeR2}**, tandem PmeR-specific operator; **P2A**, picornavirus-derived ribosome skipping sequence (N'-GATNFSLLKQAGDVEENPGP-C') optimized for bicistronic expression in mammalian cells; **pA**, polyadenylation signal; **PCR**, polymerase chain reaction; **P_{CRE2}**, synthetic mammalian promoter containing six CRE repeats; **P_{CREm}**, modified P_{CRE} variant; **P_{cFOS},** synthetic mammalian promoter containing three c-fos tandem repeats; **PEST,** peptide sequence rich in proline, glutamic acid, serine and threonine; **P_{ETR2},** erythromycin-responsive promoter; **P_{hCMV}**, human cytomegalovirus immediate early promoter; **$P_{hCMV*-1}$**, tetracycline-responsive promoter ($tetO_7$-$P_{hCMVmin}$); **$P_{hEF1\alpha}$**, human elongation factor 1α promoter; **PmeR**, *Pseudomonas syringae pathovar tomato* DC3000-derived multidrug efflux pump repressor; **PMS**, PmeR-derived paraben-mediated mammalian transsilencer (PmeR-KRAB); **P_{PMS}**, paraben-inducible promoter (P_{SV40}-O_{PmeR2}); **P_{NFAT1}**, synthetic mammalian promoter containing three tandem repeats of a human IL2 NFAT-binding site (($NFAT_{IL2}$)$_3$-P_{min}; **P_{NFAT3}**, synthetic mammalian promoter containing five tandem repeats of a IL4-derived NFAT-binding site; **P_{NFAT4}**, synthetic mammalian promoter containing seven tandem repeats of a IL4-derived NFAT-binding site **P_{NFAT5}**, synthetic mammalian promoter containing nine tandem repeats of a IL4-derived NFAT-binding site; **P_{SV40}**, simian virus 40 promoter; **P_{T7}**, promoter

activated by the T7 bacteriophage RNA polymerase; **PuroR**, gene conferring puromycin resistance; **SB100X**, optimized Sleeping Beauty transposase; **SEAP**, human placental secreted alkaline phosphatase; **TetR**, *Escherichia coli* Tn10-derived tetracycline-dependent repressor of the tetracycline resistance gene; **$tetO_7$**, TetR-specific heptameric operator sequence; **TurboGFP:dest1**, PEST-tagged TurboGFP variant (Evrogen).

Table S8. FDA-approved drugs used in this manuscript

	**Cat. No.**	**Source**
Amitriptyline hydrochloride	A8404	Sigma-Aldrich, Buchs, Switzerland
Nifedipine	N7634	Sigma-Aldrich, Buchs, Switzerland
Tetracaine hydrochloride	T7508	Sigma-Aldrich, Buchs, Switzerland
Benidipine hydrochloride	B-120	Alomone Labs, Jerusalem, Israel
Diltiazem hydrochloride	D-135	Alomone Labs, Jerusalem, Israel
Flunarizine dihydrochloride	F-110	Alomone Labs, Jerusalem, Israel
Isradipine	I-100	Alomone Labs, Jerusalem, Israel
Nicardipine HCl	N-125	Alomone Labs, Jerusalem, Israel
Nimodipine	N-150	Alomone Labs, Jerusalem, Israel
Verapamil HCl	V-100	Alomone Labs, Jerusalem, Israel
Lidocaine	L-105	Alomone Labs, Jerusalem, Israel

Supporting References

Auslander, S., Auslander, D., Muller, M., Wieland, M., and Fussenegger, M. (2012). Programmable single-cell mammalian biocomputers. Nature *487*, 123-127.

Bell, T.J., Thaler, C., Castiglioni, A.J., Helton, T.D., and Lipscombe, D. (2004). Cell-specific alternative splicing increases calcium channel current density in the pain pathway. Neuron *41*, 127-138.

Helton, T.D., Xu, W., and Lipscombe, D. (2005). Neuronal L-type calcium channels open quickly and are inhibited slowly. The Journal of neuroscience : the official journal of the Society for Neuroscience *25*, 10247-10251.

Lin, Y., McDonough, S.I., and Lipscombe, D. (2004). Alternative splicing in the voltage-sensing region of N-Type CaV2.2 channels modulates channel kinetics. Journal of neurophysiology *92*, 2820-2830.

Muller, M., Auslander, S., Spinnler, A., Auslander, D., Sikorski, J., Folcher, M., and Fussenegger, M. (2017). Designed cell consortia as fragrance-programmable analog-to-digital converters. Nature chemical biology *13*, 309-316.

Saxena, P., Heng, B.C., Bai, P., Folcher, M., Zulewski, H., and Fussenegger, M. (2016). A programmable synthetic lineage-control network that differentiates human IPSCs into glucose-sensitive insulin-secreting beta-like cells. Nature communications *7*, 11247.

Wang, H., Xie, M., Charpin-El Hamri, G., Ye, H., and Fussenegger, M. (2018). Treatment of chronic pain by designer cells controlled by spearmint aromatherapy. Nat Biomed Eng *2*, 114-123.

Wang, H., Ye, H., Xie, M., Daoud El-Baba, M., and Fussenegger, M. (2015). Cosmetics-triggered percutaneous remote control of transgene expression in mice. Nucleic acids research *43*, e91.

Xie, M., Ye, H., Wang, H., Charpin-El Hamri, G., Lormeau, C., Saxena, P., Stelling, J., and Fussenegger, M. (2016). beta-cell-mimetic designer cells provide closed-loop glycemic control. Science *354*, 1296-1301.

Xu, W., and Lipscombe, D. (2001). Neuronal Ca(V)1.3alpha(1) L-type channels activate at relatively hyperpolarized membrane potentials and are incompletely inhibited by dihydropyridines. The Journal of neuroscience : the official journal of the Society for Neuroscience *21*, 5944-5951.

Ye, H., Daoud-El Baba, M., Peng, R.W., and Fussenegger, M. (2011). A synthetic optogenetic transcription device enhances blood-glucose homeostasis in mice. Science *332*, 1565-1568.
